# Efficient Recycling of PET-PE Multilayer Packaging Materials Based on Enzymatic Depolymerization of PET

**DOI:** 10.1021/acssuschemeng.4c09388

**Published:** 2025-05-29

**Authors:** Daan M. van Vliet, Jurgen J. Mateman, Rick H.A.M. van de Vondervoort, Antoine P. H. A. Moers, Lucas Collazo, Ana Mencher, Marc W. T. Werten, Shanmugam Thiyagarajan, Arno Cordes, Christian Sonnendecker, Eggo U. Thoden van Velzen, Rosa Doménech-Mata, Juan Antonio Tamayo-Ramos, Mattijs K. Julsing, Tom A. Ewing

**Affiliations:** † Wageningen Food & Biobased Research, 4508 Wageningen University & Research, Bornse Weilanden 9, Wageningen 6708 WG, Netherlands; ‡ 202667 ITENE, Carrer d’Albert Einstein, 1, Paterna, Valencia 46980, Spain; § ASA Specialenzyme GmbH, Am Exer 19C, Wolfenbüttel 38302, Germany; ∥ Institute of Analytical Chemistry, 9180 Leipzig University, Leipzig 04103, Germany

**Keywords:** polyester hydrolase, multilayer, PET-PE, recycling, enzymatic hydrolysis, rPET

## Abstract

The transition to a sustainable, circular economy requires more plastic waste to be recycled into high-quality recycled plastics. However, it is challenging to recycle mixed waste fractions or common multilayer materials by using current mechanical recycling technology. Enzymatic hydrolysis potentially offers a solution because of its mild conditions and selectivity. In this study, we show that polyester hydrolases can be applied to recycle PET-PE multilayer packaging waste without costly amorphization pretreatment. Polyester hydrolases were produced by recombinant Pichia pastoris yeast and used to efficiently depolymerize the PET layer of PET-PE multilayer trays. High yields were obtained at laboratory scale with unpurified enzyme and high PET-PE loading (10–20% w/w PET-PE, ≥94% PET depolymerization, and ≥80% terephthalic acid recovery). The enzymatic reaction was scaled up to 4.5 kg of PET-PE production waste. After depolymerization (≥95% PET depolymerized), terephthalic acid was isolated and repolymerized into rPET. The remaining PE layer was recovered, treated with an alkaline cleaning step to remove residual PET contamination, and successfully reprocessed into rPE films with similar properties to virgin low-density PE. This study demonstrates the applicability of enzymatic hydrolysis for the recycling of PET-PE multilayer materials and highlights its general potential for the recycling of polyesters in mixed post-consumer waste.

## Graphical abstract

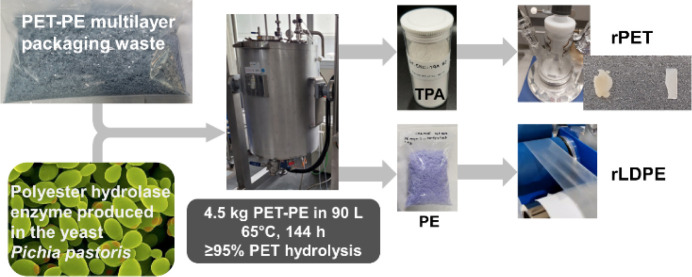

## Introduction

The accumulation of nonrecyclable plastic waste is a major societal and environmental issue. Global plastics production stood at 460 Mt in 2019, and while recycling rates are steadily increasing, the majority of plastic waste is still incinerated, landfilled, or ends up in the environment.^1^ It is imperative to increase plastic recycling rates both to limit environmental pollution and to harness plastic waste as a feedstock for a circular economy. Mechanical recycling is the most energy-efficient recycling process but only renders high-quality recycled plastics when highly pure feedstocks are used.^2^ Plastic wastes are by definition not pure, and in many commercial packages, multiple materials are used that cannot be separated mechanically.^3,4^ This issue is most obvious in the recycling of multilayer packaging materials, which consist of multiple different polymers bonded together to obtain better properties. A well-known example is polyethylene terephthalate-polyethylene (PET-PE) trays, which are typically used to package food products such as meat, cheese, and fish.^4^ Direct mechanical recycling of multilayer packaging materials leads to products with poor mechanical and optical properties due to the immiscibility of the constituent polymers, while separation of the individual polymer layers is challenging.^5^

Another possible solution is the use of chemical recycling technologies, such as pyrolysis and solvolysis, through which polymers are broken down into usable smaller molecules.^6^ In pyrolysis, waste is decomposed at high temperatures in the absence of oxygen. Pyrolysis can be used to process highly heterogeneous waste streams. However, in most cases, heterogeneous mixtures of molecules are obtained, necessitating complex upgrading steps to transform them into pure building blocks for further chemical conversions.^7^ For polymers containing hydrolyzable bonds, such as polyesters or polyamides, solvolysis processes provide an interesting option.^8^ Here, a solvent such as water, methanol, or ethylene glycol is used to degrade the polymer, typically in the presence of a catalyst. Solvolysis yields (derivatives of) the monomers from which the polymer was synthesized, which can be used to synthesize new polymers with relatively little further processing. This type of process is referred to as “back-to-monomer” recycling. For example, mild alkaline hydrolysis of PET has been studied as a recycling technology for PET-PE trays, obtaining degradation yields of approximately 60%.^9^

A specific type of solvolysis technology for the recycling of polyesters is enzymatic hydrolysis. Various polyester hydrolase enzymes are known to hydrolyze polyesters such as PET.^10–12^ Enzymatic processes for the hydrolysis of PET typically occur at relatively low temperatures in the range of 65–70 °C, in aqueous reaction media, and at mildly alkaline pH.^11,13^ For pure PET, high degrees of depolymerization (over 90%) can be achieved, and methods have been developed for the isolation and repolymerization of the monomer terephthalic acid (TPA).^13^ Enzymatic processes are potentially very interesting for processing waste streams containing multiple polymers, as polyester hydrolases selectively hydrolyze polyesters while leaving other polymers intact. Proof-of-principle for the selective hydrolysis of PET in packaging materials containing PET in combination with PE or polyamide was reported by Gamerith et al.^14^ at a scale of 50 mg, yielding 1.5 g/L TPA.

In this study, we demonstrate that polyester hydrolase enzymes can be applied in a process for the recycling of PET-PE packaging waste at up to 200 g/L plastic loading. We used the thermostable polyester hydrolases leaf-branch compost cutinase (LCC) and polyester hydrolase Leipzig 7 (PHL7) as biocatalysts. For cost-effectiveness, they were produced in the production platform Pichia pastoris and used in their crude form, which at high loadings caused inhibition of LCC but not of PHL7. Following enzymatic PET hydrolysis, the remaining PE layer was recovered, and the TPA monomer was isolated from the reaction mixture. The enzymatic reaction was scaled up to treat 4.5 kg of post-industrial PET-PE production scrap. Subsequently, TPA was isolated and repolymerized into rPET. The remaining PE layer was shown to be processable into thin low-density PE (LDPE) films. Our results highlight the practical potential of processes employing hydrolytic enzymes for recycling complex materials composed of multiple polymers.

## Experimental Section

Amorphous PET film of 0.25 mm thickness (Gf-PET) was acquired from Goodfellow (Huntingdon, UK). PET-PE trays were obtained from Termoformas (Alicante, Spain; Figure S1A–C). Post-industrial PET-PE scrap (trimming waste from PET-PE sheet production) was obtained from Aliplast SPA (Treviso, Italy; Figure S1D).

For the production of polyester hydrolases LCC and PHL7, with and without hexahistidine tags, Pichia pastoris strain GS115 was transformed using vector pPIC9 (Invitrogen; Waltham, MA, US). Methanol fed-batch fermentations of P. pastoris were performed in 2.5 L BioFlo 3000 Fermenters (New Brunswick Scientific) using minimal basal salts medium^15^ and methods described previously.^16^ Cells were separated from the broth via centrifugation and microfiltration. Scaled-up production of PHL7 was performed by methanol fed-batch fermentation of P. pastoris in a 300 L bioreactor, after which the enzyme-containing supernatant was isolated by centrifugation and concentrated by ultrafiltration. Purified hexahistidine-tagged enzymes were obtained through affinity chromatography using a HisPur Ni-NTA resin (Thermo Scientific). Enzyme purity was assessed by SDS-PAGE.^16^ Protein glycosylation was determined by SDS-PAGE with periodic acid-Schiff (PAS) glycan staining.^17^ Purified LCC produced in Escherichia coli was obtained as described previously.^12^ Esterase activity was measured by an assay using 1 mM *p*-nitrophenyl butyrate as a substrate and monitoring the absorbance of the hydrolysis product *p*-nitrophenol at 405 nm.

The degradation of plastics by polyester hydrolases was assessed by incubating approximately 45 mg of PET­(−PE) with 25 μg of polyester hydrolase in 1 M sodium phosphate buffer (pH 8.0, 1.5 mL) at 70 °C for 24 h while shaking, unless mentioned otherwise. The residual PET­(−PE) was washed, dried, and weighed to determine weight loss.

For PET hydrolysis in bioreactors, PET­(−PE) was milled to particles of <16 mm using an IKA MultiDrive. Initial reactions were conducted with a starting volume of 1.0 L and a plastic loading of 10 g/L in 3.6 L Labfors bioreactors (Infors, Bottmingen, Switzerland). Subsequent reactions at higher plastic loadings were conducted in 0.5 L Multifors bioreactors (Infors). Reactions with plastic loadings of 12.5–25 g/L were conducted with a starting liquid volume of 200–400 mL and 5.0 g of plastic. Reactions with plastic loadings of 100–200 g/L were conducted with a total starting volume of 200 mL and 20.0–40.0 g of plastic. Bioreactors were loaded with Milli-Q water or buffer solution, set to the desired temperature, crude polyester hydrolase was added, and the reaction was started by adding the plastic. Reactions were stirred at 250 rpm using a marine impeller. The pH was maintained at 8.0 by the addition of 2 M NaOH.

For pilot-scale PET hydrolysis, 4.5 kg of PET-PE suspended in 1 M NaCl and 0.2 M sodium phosphate was treated with crude PHL7 in a 120 L reactor (Figure S2, Órbita Ingeniería, Valencia, Spain). Enzyme was added to the plastic suspension, giving a reaction volume of 90 L (plastic loading of 50 g/L), and the mixture was stirred at 375 rpm and 65 °C for 144 h. The pH was maintained at 8.0 by the addition of 12 M NaOH.

After the reaction, residual solids were collected by filtration, washed, dried, and weighed. TPA was precipitated through the addition of sulfuric acid, recovered by filtration, washed, and dried. To obtain high-purity PE from the residual solid fraction, it was treated via alkaline hydrolysis. Solids were suspended in 2.5 M NaOH containing 12.5 g/L methyltrioctylammonium bromide as a phase-transfer catalyst and incubated for 16 h at 95 °C while gently shaking. The cleaned PE was obtained by filtration, washed with demineralized water and acetone, and dried. The PE was processed using a lab-scale twin-screw extruder MC 15 (Xplore Instruments, Sittard, The Netherlands) coupled to a winding system to obtain PE films (Figure S3). Crude residual solids were processed at 260–270 °C with a residence time of 3 min, while PE cleaned by alkaline hydrolysis was processed at 190–200 °C with a residence time of 4 min. Recovered terephthalic acid was repolymerized to rPET by reacting it with ethylene glycol (12 equiv) for 4.5 h at 200 °C under N_2_, followed by 2.5 h at 265 °C under 0.01 mbar vacuum in the presence of germanium­(IV) oxide (0.16% w/w TPA).

For the analysis of PET­(−PE), gel-permeation chromatography (GPC), differential scanning calorimetry (DSC), and Fourier transform infrared spectroscopy (FTIR) were performed as described previously.^18^ PE was analyzed with respect to density (ISO 1183-1), melt-flow index (MFI; ISO1133-1), tensile properties (ISO 527), and diffuse opacity and color. Aromatic PET monomers were quantified by reversed-phase high-performance liquid chromatography (RP-HPLC). For proton nuclear magnetic resonance spectroscopy (^1^H NMR), TPA samples were dissolved in deuterated dimethyl sulfoxide. PET samples were dissolved in a 6:1 (v/v) deuterated chloroform:trifluoroacetic acid.

A detailed description of the Materials and Methods can be found in the Supporting Information.

## Results and Discussion

Three different PET-PE packaging materials were obtained (Figure S1) and their material properties were analyzed. They included a transparent PET-PE tray (PET-PE#1), a transparent PET-PE clamshell (PET-PE#2), and a white opaque PET-PE tray (PET-PE#3). These materials were analyzed by GPC, DSC, and FTIR. Gf-PET was analyzed in the same manner, as was Gf-PET milled to particles of <16 mm. FTIR revealed that, for the PET-PE materials, one side of the plastic displayed an FTIR spectrum characteristic of PET, while the other side displayed an FTIR spectrum characteristic of PE (Figures S4and S5). This demonstrates that the PET layer was at the surface of the materials and therefore accessible for enzymatic hydrolysis. GPC showed that the molar mass of PET in PET-PE#1 and PET-PE#2 was similar to that of Gf-PET, while in PET-PE#3 it was somewhat lower (Table 1). PET-PE#1 and PET-PE#2 were mainly composed of PET (96% and 97%, respectively), while PET-PE#3 had a PET content of 82% (Table 1). DSC revealed that in all of the studied materials, the PET was predominantly in the amorphous form (Figures S6 and S7). The analysis of the milled Gf-PET revealed that milling did not affect any of the studied properties.

**Table 1:** Properties of PET and PET-PE Materials Used in this Study^a^

Sample	Material type	Thickness (mm)	PET content (% w/w)*	*M*_w_ PET (10^3^ g/mol)	PET degree of crystallinity (%)
Gf-PET	Sheet	0.25	100.6 ± 1.3	45.4 ± 0.0	3
Gf-PET milled	Sheet	0.25	100.9 ± 2.3	44.9 ± 0.3	1
PET-PE #1	Multilayer Tray	0.25	95.6 ± 2.9	48.5 ± 8.0	0
PET-PE #2	Multilayer Clamshell	0.32	96.6 ± 2.5	47.8 ± 0.7	0
PET-PE #3	Multilayer Tray	0.60	82.1 ± 2.0	38.0 ± 6.0	0

a ±: Standard deviation of two replicates*. As determined by GPC. The PET content of 101%, determined for Gf-PET and Gf-PET milled, is within the margin of error expected for a material consisting completely of PET.

Two polyester hydrolases were used in this study: LCC, a benchmark enzyme active in unbuffered water,^13^ and PHL7, which showed higher specific activity toward PET but requires considerable ionic strength.^12^ They were produced in P. pastoris, a yeast highly suited for the production of recombinant proteins as it yields high concentrations of secreted product (Figure S8). Secretory enzyme production simplifies downstream processing compared to intracellularly produced proteins. The enzymes were purified and analyzed by SDS-PAGE, displaying major bands between 28 and 38 kDa (Figure S9, theoretical: 28–29 kDa). The smearing observed above the bands and the higher-than-expected molar mass indicated that the proteins are glycosylated, as was reported previously for LCC.^19^ This was confirmed by glycan staining (Figures S10 and S11). Shirke *et al.* reported that glycosylation of LCC improved stability against thermal aggregation, improving PET hydrolysis at 70 °C.^19^ In a concurrent study focusing on PHL7 glycosylation, increased thermostability was also observed, along with slightly decreased activity toward PET at lower temperatures.^20^ Purified LCC produced in P. pastoris displayed a specific esterase activity of 98 U/mg (±13 U/mg standard error [SE]). For purified LCC produced in E. coli, we found 107 U/mg (±9 U/mg SE). It was assumed that the His-tag did not influence the specific activity. Based on the typical esterase activity of 12–19 U/mL in P. pastorisfermentation supernatants (*n* = 5), a product titer of 0.12–0.19 g/L was estimated for LCC. PHL7 showed a lower specific esterase activity (23 U/mg, ± 1 U/mg SE), but a higher product titer of 2.1 g/L (±0.23 standard deviation [SD], *n* = 1, *N* = 3).

To evaluate whether the PET layer of the PET-PE materials is accessible to polyester hydrolases, they were subjected to degradation using purified enzymes from P. pastoris. Degradation of the PET-PE materials was compared with that of Gf-PET, a substrate known to be readily degradable (Figure 1A). Under the applied reaction conditions, full degradation was not expected, even for Gf-PET. Therefore, these results serve as a measure of the relative rate of depolymerization of PET and not of the maximum degree of depolymerization that can be achieved. For all materials and both enzymes, significant depolymerization of PET was observed (Figure 1A). This demonstrates that the amorphous nature of the PET in these materials allows it to be readily degraded. The PET in PET-PE multilayers was hydrolyzed to a somewhat lower extent than Gf-PET. The lower accessible surface area of PET in the multilayer materials (as one side of the material is PE) may explain this.

**Figure 1:**
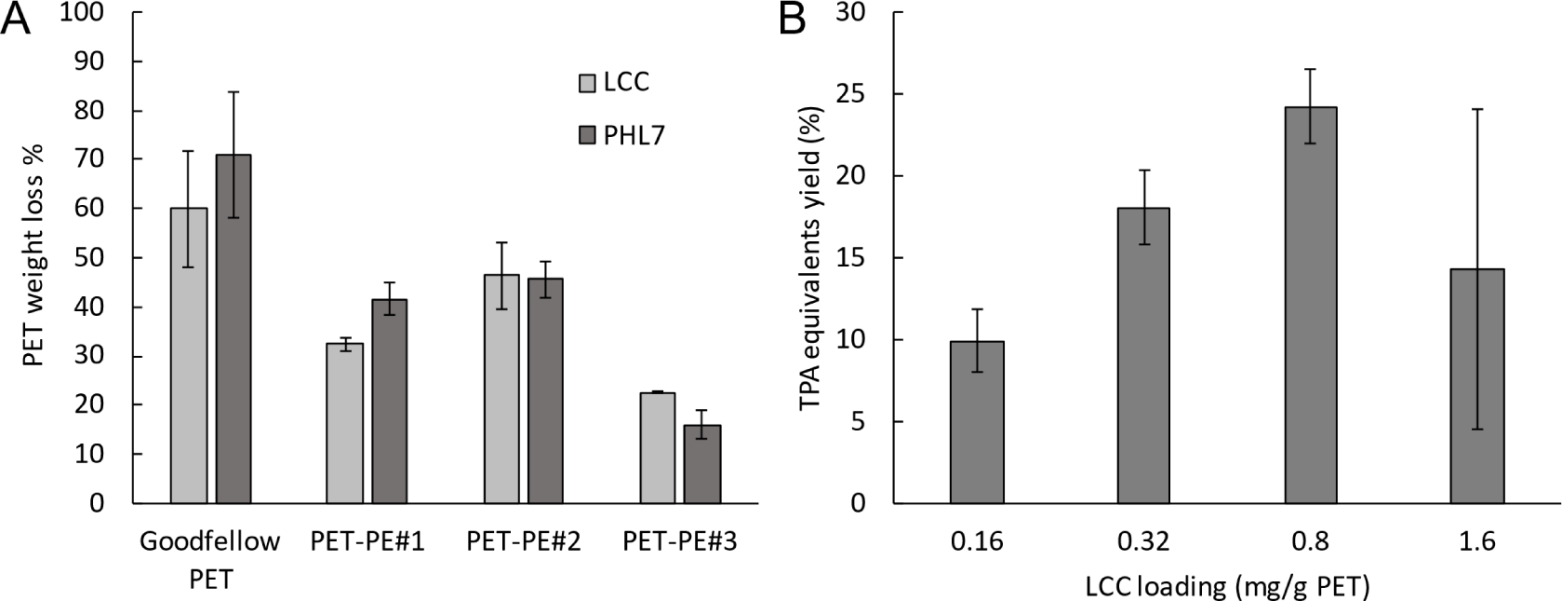
PET hydrolysis assay results after 24 h of incubation: (A) comparing different PET­(−PE) types and (B) different enzyme loadings of crude LCC. Assessment of optimal crude LCC loading was done using Gf-PET at 65 °C, followed by determination of the concentration of produced TPA equivalents by UV spectroscopy at 240 nm wavelength.

We then set out to develop an efficient process for the hydrolysis of PET-PE#1 and PET-PE#2 layers in a bioreactor. We first analyzed the enzyme loading required to obtain optimal PET hydrolysis for the model substrate Gf-PET in small-scale tests. The use of purified enzymes in such a process is undesirable due to the costs associated with enzyme purification. Therefore, we also tested whether crude P. pastoris fermentation supernatant could be applied directly. We initially focused our efforts on LCC, as it had been shown to maintain its activity in unbuffered water, an interesting property for the development of a cost-effective recycling process.^13^ An optimal hydrolysis of Gf-PET by crude LCC was obtained at a loading of 0.8 mg_LCC_/g_PET_ (Figure 1B). Based on these results, an enzyme loading of 0.8 mg_LCC_/g_PET‑PE_ was initially employed in the hydrolysis process.

Next, we subjected PET in PET-PE#1 and PET-PE#2 to hydrolysis by crude LCC in a pH-controlled bioreactor setup and compared the degradation efficiency to that of Gf-PET (see Table S1 for a summary of all laboratory- and pilot-scale reactions). As the use of buffer salts is unattractive from an economic point of view, we performed the depolymerization in ultrapure water with the pH maintained at 8.0. The temperature of the reaction mixture was set to 70 °C, which provides a good balance between catalytic efficiency and PET recrystallization.^11^

The PET in the multilayer materials was depolymerized more slowly than Gf-PET, as evidenced by the slower release of soluble aromatic products (Figure 2A). This was confirmed by determining the weight loss of the materials after the reactions. For Gf-PET, essentially complete depolymerization (less than 1% of the starting weight recovered as solids, with a 99% aromatic monomer yield) was observed after 43 h. A constant level of esterase activity in solution over time indicated that LCC was stable and had been added in sufficient excess (Figure S12). For PET-PE#1 and PET-PE#2, PET depolymerization after 67 h was 89% and 84%, respectively. HPLC analysis indicated aromatic monomer yields that were similar, although slightly higher (95% and 91%, respectively), possibly due to the evaporation of some liquid from the reaction medium. Three monomeric aromatic products were formed from all substrates used (Figure 2B): the desired product TPA, which constantly increased in concentration; the intermediate MHET, which accumulated only transiently due to subsequent hydrolysis to TPA; and the TPA isomer isophthalic acid, which constantly accumulated in minor amounts compared to the other products. Isophthalic acid is commonly added as a comonomer during PET production and therefore likely originates from the PET substrate.^21,22^

**Figure 2:**
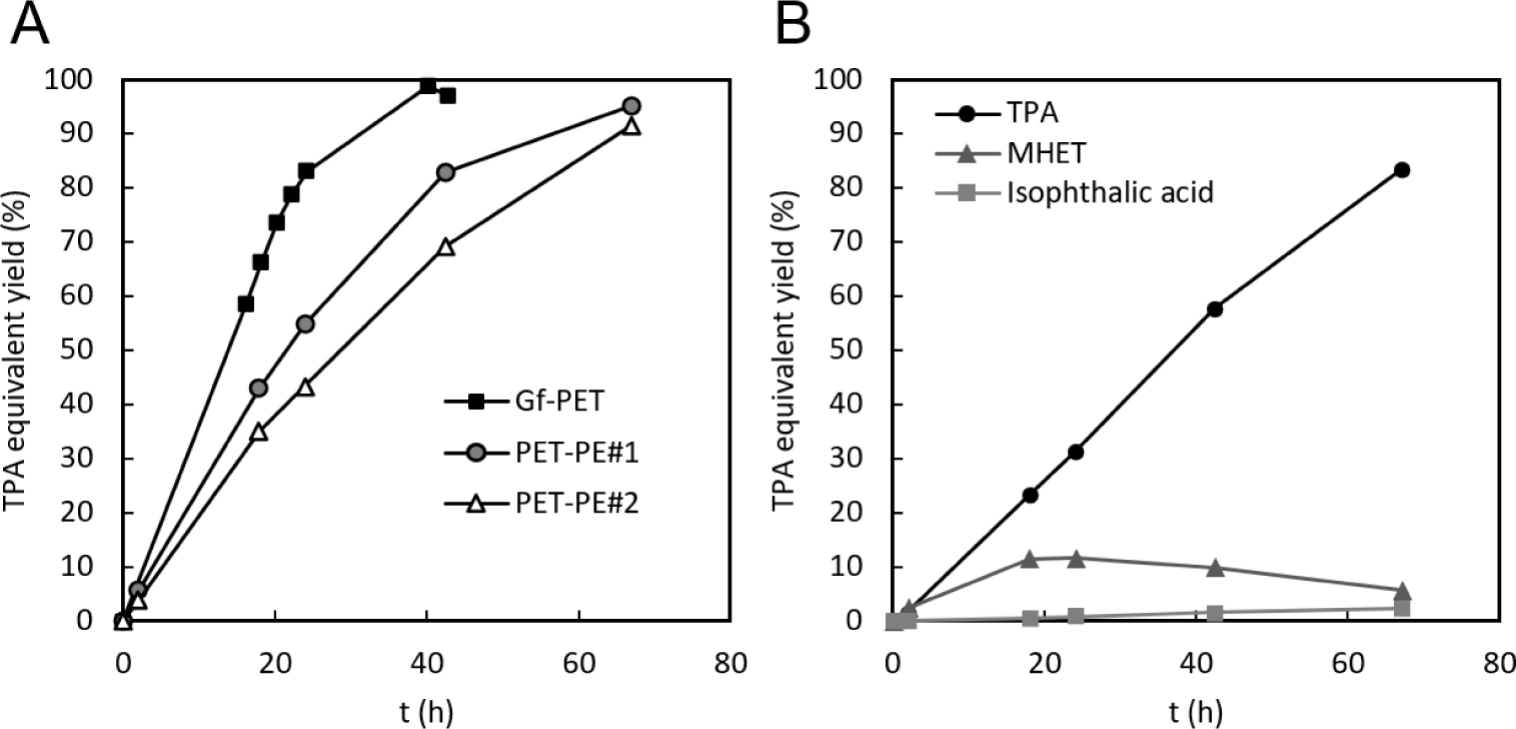
Accumulation of dissolved aromatic monomers over time resulting from enzymatic PET hydrolysis of 10 g of PET­(−PE) with crude LCC in bioreactors, at 10 g_PET(−PE)_/L, 0.8 mg_LCC_/g_PET(‑PE_ (75 U/g_PET(−PE)_), 70 °C, and pH 8. (A) Total TPA equivalents, consisting of TPA, MHET, and isophthalic acid concentrations summed. (B) Individual aromatic monomer production profile with PET-PE#2 as feedstock, expressed as percentage of the total maximal TPA equivalent yield. Very similar profiles were observed with Gf-PET or PET-PE#1.

The solids remaining after the reactions with PET-PE#1 and PET-PE#2 were isolated by filtration (Figure S13). FTIR analysis revealed that PET and PE were both still present, with spectra similar to those obtained before enzymatic treatment (Figure S14). This indicates that no significant chemical changes had occurred in the PE layer. The residual PET content was 64–76% w/w, as determined by GPC, with no significant difference in average molar mass from the starting material (Figure S15). Interestingly, this residue had become white and opaque (Figure S13), whereas the starting material was colorless and transparent. This indicates that the residual PET has recrystallized during the reaction. This was confirmed by DSC, which revealed that the PET crystallinity had increased from <1 to 18–21% (Figure S16). Recrystallization of PET may be prevented by lowering the reaction temperature. To test whether this could lead to a higher degree of PET depolymerization, reactions were repeated at 65 and 70 °C with a plastic loading of 12.5 g/L and an incubation time of 115 h. A higher degree of depolymerization was observed at 65 °C compared to 70 °C, with >95% removal of the PET layer for both materials (Figure S17). The amount of PET monomers released was in good agreement with the weight loss (Figure S17). These experiments demonstrate that enzymatic hydrolysis allows us to depolymerize the PET layer of PET-PE multilayer materials, yielding a reaction mixture containing PET monomers and a residual plastic fraction containing the PE layer.

We then set out to increase the plastic loading, an important parameter for an economically viable recycling process.^23^ When increasing the plastic loading to 100 g/L or above, the reproducibility of the depolymerization process was poor when applying LCC-containing P. pastoris fermentation supernatants, and the depolymerization rate and final degree of depolymerization were typically lower than expected. This could be related to the presence of (unidentified) inhibiting substances in the P. pastoris fermentation supernatant. This is supported by observations in small-scale degradation assays (Figure 1B), where a reduced degradation efficiency was observed when increasing the enzyme loading to over 0.8 mg/g_PET_. The large amounts of supernatant required to maintain the required enzyme loading per gram of plastic at higher plastic loadings might lead to higher concentrations of the inhibitory compound being present, slowing the reaction down. The presence of an inhibitory component in the fermentation medium of P. pastoris was confirmed by spiking small-scale assays of PET degradation by purified LCC with fermentation supernatant from a different P. pastoris strain (i.e., a strain not expressing a polyester hydrolase), leading to strongly decreased depolymerization of PET. Similarly, Ideonella sakaiensis PETase showed inhibition by soluble components of P. pastoris medium, possibly metal ions.^24^

To enable efficient and reproducible depolymerization of PET from PET-PE multilayers at plastic loadings of ≥100 g/L, we tested the performance of the PHL7 polyester hydrolase. PHL7 was produced at higher titers in P. pastoris. This means that significantly lower volumes of PHL7-containing fermentation supernatant must be applied to achieve the desired enzyme loading per gram of plastic, providing a way to limit inhibition from components of the fermentation medium. This was verified experimentally in small-scale Gf-PET hydrolysis, showing efficient PET hydrolysis at high enzyme loadings of 2–20 mg_PHL7_/g_PET_ (Figure S18). A disadvantage of using PHL7, as compared to LCC, is that PHL7 does not retain its activity in a purely aqueous environment, necessitating the addition of salts such as NaCl or sodium phosphate (NaP_i_) to the reaction medium. In reactions at 70 °C using 4 mg_PHL7_/g_PET_, Gf-PET was hydrolyzed in 1 M NaP_i_, but no hydrolysis was detected at <0.1 M NaP_i_. PET in the form of PET-PE#2 could also be hydrolyzed in the presence of 1 M NaCl in the absence of NaP_i_ (Figure S19), but the rate was 3-fold lower (0.17 versus 0.47 g L^–1^ h^–1^). As a compromise between efficiency and cost-effectiveness, we continued the reactions in 0.2 M NaP_i_ supplemented with 1 M NaCl.

Fermentation of the supernatant from P. pastoris expressing PHL7 was employed for the depolymerization of PET-PE#2 at plastic loadings of 100 and 200 g/L. A modest enzyme loading of 0.75 mg/g_PET‑PE_ (17 U/mg_PET‑PE_) was used, similar to the previously used LCC loading (0.8 mg/g) and to the purified PHL7 loading used in other studies (0.6 mg/g). ^12^ A temperature of 65 °C was used to suppress PET recrystallization. Based on weight loss, after 190 h, PET depolymerization degrees of 94% and 96% were achieved for plastic loadings of 100 and 200 g/L, respectively. The production of PET monomers slowed down over the course of the reactions (Figure 3A), starting at 1.1 and 2.3 g L^–1^ h^–1^ during the first 24 h and reaching overall rates of 0.4 and 0.7 g L^–1^ h^–1^, respectively. The overall specific rate was 5 g g_PHL7_^–1^ h^–1^ in both cases. Yields of monomers determined by HPLC were slightly lower than the depolymerization extent, at 87% and 84%, respectively (Figure 3B). The cause of this apparent discrepancy is unclear, but it could be due to the precipitation of PET monomer salts against the reactor walls. TPA was isolated from the hydrolyzate at yields of 80% and 81% for the reactions at 200 g/L and 100 g/L plastic, respectively (Figure S20), analyzed by RP-HPLC and ^1^H NMR (Figure S21) and shown to have a purity of 95 ± 1% and 94 ± 1% w/w, respectively, with the only observed impurities being 3% w/w isophthalic acid and 3% w/w MHET, which are both not problematic for subsequent repolymerization.^25,26^ Remaining solid plastic was also recovered from the reaction (Figure S20). GPC showed 35–45% w/w PET content and no significant change in the PET molar mass distribution (Table S2). Weight loss following alkaline PET hydrolysis indicated the presence of 39–47% w/w PET, confirming the GPC result. DSC showed that the remaining PET was amorphous (Figure S22).

**Figure 3:**
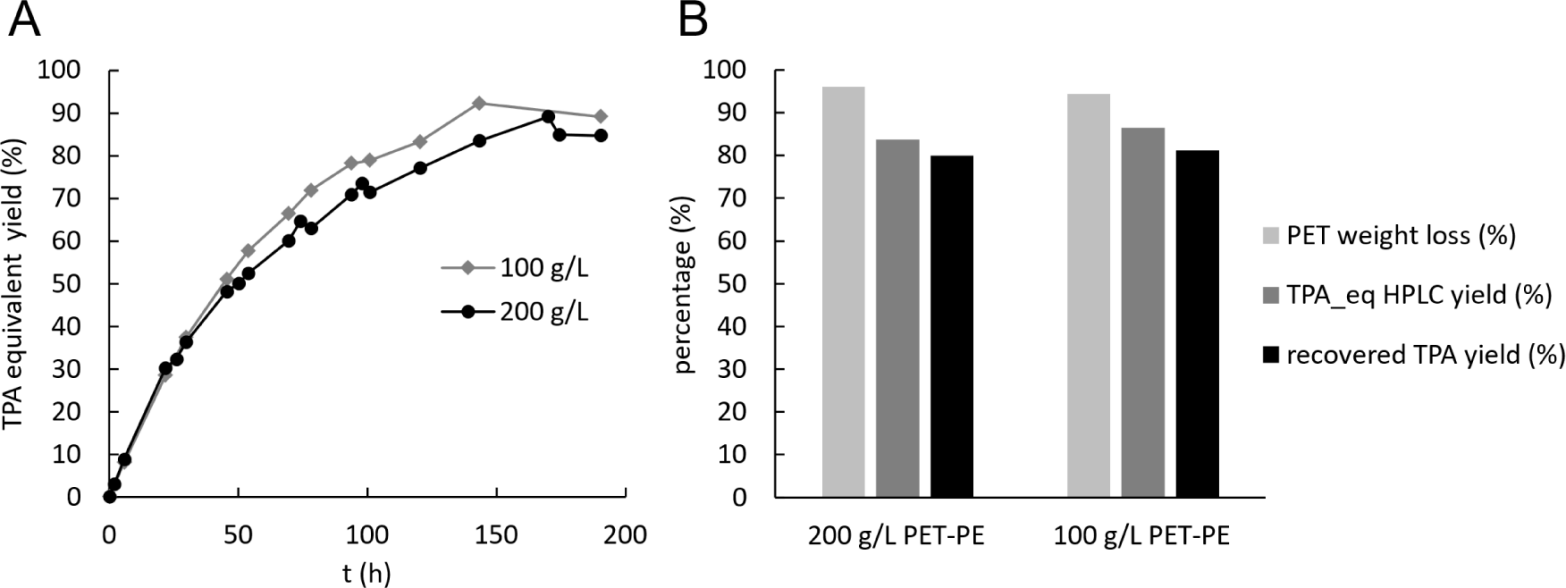
PET hydrolysis by crude PHL7 at high PET-PE#2 loadings. (A) Yield of aromatic PET monomers (TPA equivalents) over time. (B) yields. “TPA_eq” is an abbreviation of TPA equivalents in solution.

To produce sufficient enzyme for scaled-up depolymerization trials, the process for the production of PHL7 was scaled up in a 300 L bioreactor. After 95 h, the culture supernatant was harvested by centrifugation and concentrated by ultrafiltration, with an activity yield of 90%, producing a preparation of 4 L containing 7.6 million units of activity at 1.9 kU/mL and 42 mg/mL protein (45 U/mg; Table S3).

Subsequently, the depolymerization process was scaled up to 4.5 kg of PET-PE. Cutting waste generated during the production of PET-PE sheets was used as the substrate. This post-industrial scrap consisted of colored flakes with dimensions of 5–8 mm (Figure S1D). The PET content of this material was 95.6%. Reactions were conducted in duplicate at a PET-PE loading of 50 g/L and at a high enzyme loading (100 U/g_PET‑PE_; 4 mg_PHL7_/g_PET‑PE_) to achieve complete PET hydrolysis. After a reaction time of 144 h, PET depolymerization degrees of 98% and 99% were achieved, as determined by measuring weight loss. When the reaction was repeated using a halved enzyme loading (50 U/g_PET‑PE_; 2 mg_PHL7_/g_PET‑PE_), a slightly lower yet still significant depolymerization degree of 95% was observed. The obtained overall depolymerization rate was 0.33–0.34 g L^–1^ h^–1^. The specific rates were 1.7 g g_PHL7_^–1^ h^–1^ for the high enzyme loading condition and 3.3 g g_PHL7_^–1^ h^–1^ for the condition with halved enzyme loading. These rates are consistent with those obtained at the laboratory scale, thereby confirming the scalability of the process. Finally, efforts were focused on assessing the recyclability of the resulting materials through two approaches. The recyclability of the PET monomers was assessed by isolating and purifying TPA to subsequently repolymerize it into recycled PET (rPET). The feasibility of mechanical recycling of the isolated PE layer was investigated by the production of a thin PE film.

Residual solids were removed from the hydrolyzate by filtration, leaving a clear solution. Acidification of the filtrate resulted in the precipitation of fine white TPA particles that were isolated as a powder after filtration, washing with water, drying, and grinding. The crude TPA was analyzed by ^1^H NMR (Figure S24), showing that the TPA only contained an impurity of 2% isophthalic acid. The (combined) purity of terephthalic and isophthalic acid was >99% based on quantitative NMR with maleic acid as an internal standard. The presence of isophthalic acid was considered beneficial, as our purpose was to repolymerize the TPA into PET, for which it is common to add a small percentage of isophthalic acid to tune the polymer properties.

A polymerization reaction was carried out to test if the obtained TPA was sufficiently pure to repolymerize into rPET. The polyesterification of crude TPA with ethylene glycol, using GeO_2_ as a catalyst, produced a product with a light-brown color (Figure S25). A minor impurity present in the isolated TPA, which we did not detect by ^1^H NMR—e.g., protein from the P. pastorisfermentation supernatant—may be responsible for this coloration. Color formation was greatly reduced by washing the crude TPA with boiling ethylene glycol prior to polymerization, resulting in an off-white polymeric product (Figure S25). The molar mass of the polymer obtained from TPA washed with ethylene glycol was similar to that of Gf-PET, while the molar mass of the polymer obtained from the crude TPA product was somewhat higher (Table 2). The ^1^H NMR spectra (Figure S26) of both polymers showed all characteristic signals of PET (signals at δ 8.13 and 4.79), but also a rather high incorporation of diethylene glycol units (signals at δ 8.09, 4.63, and 4.11),^21^ which is probably due to the large excess of ethylene glycol used in our polymerization procedure. Further optimization and scale-up of the polymerization procedure—e.g., demonstrating it also works effectively at substrate loadings over 50 g/L—were beyond the scope of this study. Based on our initial findings, the successful development of a process in which TPA from enzymatically depolymerized PET-PE material is recycled into high-quality recycled PET seems promising.

**Table 2:** Color and Molar Mass (*M*_n_ and *M*_w_, Determined by GPC) of Synthesized rPET Samples^a^

Sample	Polymer color	*M*_n_ (10^3^ g/mol)	*M*_w_ (10^3^ g/mol)	*M*_w_/*M*_n_
rPET from crude TPA	Light-brown	27 ± 3	62 ± 9	2.4 ± 0.3
rPET from TPA washed with hot ethylene glycol	Off-white	21 ± 4	48 ± 1	2.3 ± 0.4
Goodfellow PET film (reference material)	Translucent	24 ± 3	48 ± 3	1.8 ± 0.2

a ± : Standard deviation across four measurements.

The thermal properties of both the crude recovered PE and PE cleaned by alkaline hydrolysis were characterized using DSC (Table S4 and Figure S27). Two prominent endothermic peaks were observed at approximately 110 and 120 °C, which can be attributed to the melting of low-density PE (LDPE). Notably, an endothermic peak at 250 °C indicated the presence of PET contamination in the crude PE samples. The density of crude PE was 0.99 g/cm^3^, while cleaned PE exhibited a lower density of 0.94 g/cm^3^. The melt flow index (MFI) of crude PE was significantly higher and less stable than that of cleaned PE, with values of 0.55 (±0.21 SD) and 0.24 (±0.03 SD) g/min, respectively. The difference in MFI may be attributed to the 10-fold higher loading weight necessary for the analysis of crude PE, but the high standard deviation for crude PE is likely attributable to PET contamination. Processing of crude PE into a film required elevated temperatures between 260 and 270 °C, presumably due to the presence of PET. In contrast, cleaned PE could be processed into a film at temperatures between 180 and 190 °C (Figure 4), which is the expected temperature range for LDPE processing.

**Figure 4:**
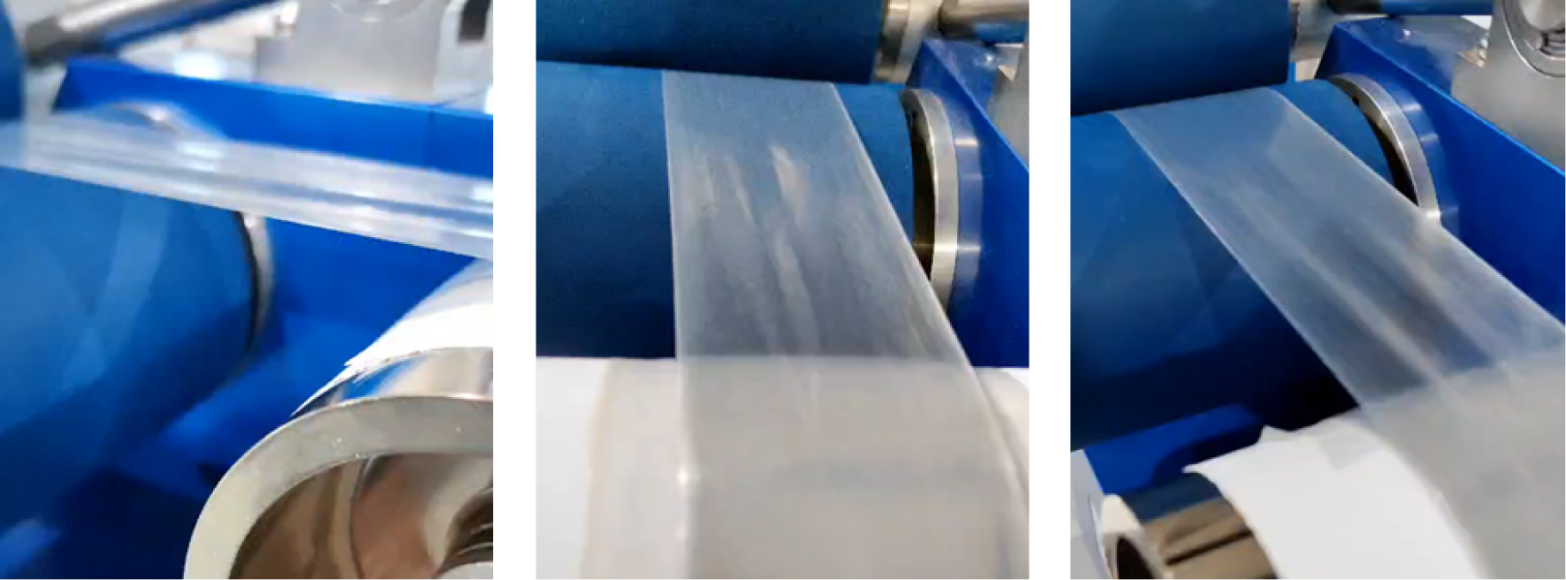
Three photographs from different perspectives of PE film produced from cleaned, recovered PE.

The film produced from crude PE showed a Young’s modulus and strain at break higher than those of the film produced from cleaned PE (Table 3). This difference can be attributed to the combined effects of greater thickness and the presence of PET in the crude material. The peak stress values for both recycled PE films were comparable (Table 3). The opacity of the film from crude PE was determined to be 8.5%, while the film from cleaned PE exhibited a lower opacity of 6.1%. Colorimetric analysis revealed that the color coordinates of both films were largely similar, although the film from crude PE showed a notably lower lightness, indicating a darker hue (Table 3). From these findings, it can be concluded that the mechanical properties of the recycled film from cleaned PE fall within the expected range for virgin LDPE films.^27^

**Table 3:** Tensile Test Results and CIELAB Color Coordinates of Recycled PE Films^a^

Sample	Thickness (mm)	Stress at peak [MPa]	Young modulus [GPa]	Strain at break [%]	L*	a*	b*
Crude PE from hydrolyzate	0.1	11.0 ± 1.4	0.40 ± 0.04	433 ± 23	69.8 ± 1.5	4.5 ± 0.1	-10.0 ± 0.5
PE from hydrolyzate after alkaline treatment	0.04	11.4 ± 2.4	0.21 ± 0.05	339 ± 62	88.0 ± 0.8	4.5 ± 0.1	-11.0 ± 0.3

a L*: lightness coordinate; a*: red–green coordinate; b*: yellow–blue coordinate; ±, standard deviation

## Conclusion

Polyester hydrolases, heterologously produced as secreted enzymes by recombinant P. pastoris, were employed in their crude, cost-effective form for the efficient depolymerization of the PET layer of PET-PE trays and post-industrial scrap from the production of PET-PE sheet, at a scale of up to 4.5 kg. Upon completion of the depolymerization process (achieving ≥ 95% removal of PET), TPA was successfully isolated and repolymerized into rPET. The residual PE layer was successfully processed into PE films, although a mild alkaline cleaning step was required to remove PET residues and provide a film with satisfactory properties. These results highlight the potential of selective enzymatic hydrolysis to be employed in the recycling of multilayer materials where one layer is subject to enzymatic hydrolysis but the other is not. In addition to clean multilayer plastic waste, such as that used in this study, this strategy provides enzymatic hydrolysis with great potential to be applied in the recycling of mixed post-consumer waste. For example, waste fractions containing significant amounts of amorphous PET waste (such as PET trays) in the presence of other plastic packaging, paper, glue, or food residues might be highly suited for processing by using enzymatic processes. More studies with such a focus will be useful in further defining the application scope of enzymatic recycling technologies.

## Supplementary Material



## Notes

Supporting Information (pdf) contains detailed materials and methods, photos of materials and experimental set-ups, FTIR, DSC, SDS-PAGE, GPC, ^1^H NMR data, and further details on selected plastic degradation experiments and scaled-up enzyme production (PDF)

## #

Organon Pharma BV, Kloosterstraat 6, 5349 AB Oss, The Netherlands
