# Supplementary material for: Efficient Recycling of PET-PE Multilayer Packaging Materials Based on Enzymatic Depolymerization of PET
Source: ACS Sustain Chem Eng. 2025 May 29;13(22):8212–9. doi: 10.1021/acssuschemeng.4c09388 (PMC12153037; doi:10.1021/acssuschemeng.4c09388)
Supplement: Supplementary file 1 [file sc4c09388_si_001.pdf]

Efficient recycling of PET-PE multilayer packaging materials based on enzymatic depolymerization of PET

Daan M. van Vliet[a], Jurgen J. Mateman[a], Rick van de Vondervoort[a,1], Antoine P.H.A. Moers[a], Lucas Collazo[b], Ana Mencher[b], Marc W.T. Werten[a], Shanmugam Thiyagarajan[a], Arno Cordes[c], Christian Sonnendecker[d], Eggo U. Thoden van Velzen[a], Rosa Doménech-Mata[b], Juan Antonio Tamayo Ramos[b] , Mattijs K. Julsing[a], Tom A. Ewing[a,*]

a. Wageningen Food & Biobased Research, Wageningen University & Research, Bornse Weilanden 9, 6708 WG Wageningen, the Netherlands
b. ITENE, Carrer d'Albert Einstein, 1, 46980 Paterna, Valencia, Spain
c. ASA Specialenzyme GmbH, Am Exer 19C, 38302 Wolfenbüttel, Germany
d. Institute of Analytical Chemistry, Leipzig University, 04103 Leipzig, Germany
1. current address: Organon Pharma BV, Kloosterstraat 6, 5349 AB Oss, The Netherlands

*Corresponding author, tom.ewing@wur.nl

28 pages, 27 supplementary figures, 4 supplementary tables

Supplementary Materials & Methods

Materials

Amorphous PET film of 0.25 mm thickness (Gf-PET) was acquired from Goodfellow (Huntingdon, UK). PET-PE trays were obtained from Termoformas (Alicante, Spain; Fig. S1ABC). Post-industrial PET-PE scrap (trimming waste from PET-PE sheet production) was obtained from Aliplast SPA (Treviso, Italy; Fig. S1D). Methanol (≥99.98%) was obtained from Biosolve B.V. (the Netherlands). All other chemicals were obtained from Sigma-Aldrich, currently Merck (Darmstadt, Germany), at the highest purity available.

Pichia pastoris strain construction

A synthetic gene encoding LCC was ordered from GenScript Biotech (Piscataway, NJ, US). The coding DNA sequence, codon-optimized for *Pichia pastoris*, was identical to the DNA sequence specified by Shirke et al. [1]. The encoded protein corresponds to UniProt accession number G9BY57 without leader sequence (residues 1-35) and with an added C-terminal hexahistidine-tag. The sequences 5'-**CTCGAG**AAAAGAGAGGCTGAAGCT-3' and 5'-TAATA**GAATTC**-3' were added to the 5' and 3' ends of the coding sequence, respectively, to allow seamless cloning into the *Xho*I and *Eco*RI sites (marked in bold face in the sequences) of vector pPIC9 (Invitrogen; Waltham, MA, US). The 5' sequence restores the encoded (Glu-Ala)$_2$ spacer that is removed from the vector when using the *Xho*I site. This spacer is commonly employed to facilitate efficient Kex2-processing of the α-mating factor prepro secretory signal. An LCC variant where the hexahistidine-tag was omitted was similarly constructed.

The PHL7 amino acid sequence used corresponds to ENA accession number LT571446 without its N-terminal Met residue and with an added C-terminal hexahistidine-tag. The corresponding gene, codon-optimized for *Pichia pastoris*, was ordered from GenScript Biotech. Sequences (underlined) were added to the 5' and 3' ends as indicated above for LCC to allow seamless cloning into vector pPIC9 via *Xho*I and *Eco*RI sites (marked in bold face):

CTCGAGAAAAGAGAGGCTGAAGCTGCCAACCCTTATGAGAGAGGACCAGACCCAACAGAGAGTTCCATTG
AGGCCGTTAGAGGACCATTTGCCGTCGCCCAGACTACCGTCTCTAGATTGCAAGCTGATGGTTTCGGTGG
TGGTACTATCTACTATCCAACTGATACTTCTCAAGGTACTTTCGGTGCTGTTGCTATTTCTCCAGGTTTC
ACTGCTGGTCAAGAATCTATTGCTTGGTTGGGTCCAAGAATTGCTTCTCAAGGTTTCGTTGTTATTACTA
TTGATACTATTACTAGATTGGATCAACCAGATTCTAGAGGTAGACAATTGCAAGCTGCTTTGGATCACTT
GAGAACTAACTCTGTTGTTAGAAACAGAATTGATCCAAACAGAATGGCTGTTATGGGTCACTCTATGGGT
GGTGGTGGTGCTTTGTCTGCTGCTGCTAATAACACTTCTTTGGAAGCTGCTATTCCTTTGCAAGGTTGGC
ATACTAGAAAGAACTGGTCTTCTGTTAGAACTCCAACTTTGGTTGTTGGTGCTCAATTGGATACTATTGC
TCCAGTTTCTTCTCATTCTGAGGCTTTCTACAACTCTTTGCCATCTGATTTGGATAAGGCTTACATGGAG
TTGAGAGGTGCTTCTCATTTGGTTTCTAACACTCCTGATACTACTACTGCTAAGTACTCTATTGCTTGGT
TGAAGAGATTCGTTGATGATGATTTGAGATACGAGCAATTCTTGTGTCCTGCCCCAGACGATTTCGCCAT
CTCCGAGTATAGAAGTACCTGTCCTTTCCATCATCATCATCATCATTAATA**GAATTC**

A PHL7 variant without hexahistidine-tag was similarly constructed, giving the following gene sequence:

CTCGAGAAAAGAGAGGCTGAAGCTGCCAACCCTTATGAGAGAGGACCAGACCCAACAGAGAGTTCCATTG
AGGCCGTTAGAGGACCATTTGCCGTCGCCCAGACTACCGTCTCTAGATTGCAAGCTGATGGTTTCGGTGG
TGGTACTATCTACTATCCAACTGATACTTCTCAAGGTACTTTCGGTGCTGTTGCTATTTCTCCAGGTTTC
ACTGCTGGTCAAGAATCTATTGCTTGGTTGGGTCCAAGAATTGCTTCTCAAGGTTTCGTTGTTATTACTA
TTGATACTATTACTAGATTGGATCAACCAGATTCTAGAGGTAGACAATTGCAAGCTGCTTTGGATCACTT
GAGAACTAACTCTGTTGTTAGAAACAGAATTGATCCAAACAGAATGGCTGTTATGGGTCACTCTATGGGT
GGTGGTGGTGCTTTGTCTGCTGCTGCTAATAACACTTCTTTGGAAGCTGCTATTCCTTTGCAAGGTTGGC
ATACTAGAAAGAACTGGTCTTCTGTTAGAACTCCAACTTTGGTTGTTGGTGCTCAATTGGATACTATTGC
TCCAGTTTCTTCTCATTCTGAGGCTTTCTACAACTCTTTGCCATCTGATTTGGATAAGGCTTACATGGAG
TTGAGAGGTGCTTCTCATTTGGTTTCTAACACTCCTGATACTACTACTGCTAAGTACTCTATTGCTTGGT
TGAAGAGATTCGTTGATGATGATTTGAGATACGAGCAATTCTTGTGTCCTGCCCCAGACGATTTCGCCAT
CTCCGAGTATAGAAGTACCTGTCCTTTCTAATA**GAATTC**

The expression vectors obtained were linearized with *Sal*I and used to transform *P. pastoris* strain GS115 via electroporation. Using colony PCR, Mut^+ transformants were selected where the vector had integrated at the *his4* locus (i.e., strains featuring normal growth on methanol).

Enzyme production and purification

Fed-batch fermentations of *Pichia pastoris* were performed in 2.5 L Bioflo 3000 fermenters (New Brunswick Scientific) using minimal basal salts medium [2] and methods described previously [3]. The pH was maintained at 5.0 during the glycerol batch phase and was increased to 5.5 – 6.0 half an hour prior to methanol induction. The methanol fed-batch phase lasted 3–4 days. The cells were separated from the broth by centrifugation (30–60 min, 12,000–16,900 x*g*), followed by microfiltration. For purification of His-tagged PET hydrolases, the cell-free fermentation supernatant was adjusted to pH 8 by addition of 1 M NaOH. After centrifugation (10,000 x*g*, 10 min), 200 mL of supernatant was mixed with an equal volume of phosphate-buffered saline (PBS, pH 7.4) containing 10 mM imidazole and incubated with 5 mL HisPur Ni-NTA resin (Thermo Scientific) for 30 min while mixing. The resin was washed five times with 10 mL PBS containing 25 mM imidazole before polyester hydrolase was eluted with three times 5 mL of PBS containing 250 mM imidazole. The eluate containing the purified polyester hydrolase was buffer-exchanged for 50 mM sodium phosphate pH 8.0 and 0.3 M NaCl using a 10 kDa Vivaspin 20 ultrafiltration unit (Sartorius) and filtered (0.2 µm pore size). Protein concentration was measured using the Pierce Bradford assay using BSA as a reference (ThermoFisher Scientific). Enzyme purity was assessed by SDS-PAGE as described previously [3]. Protein glycosylation was determined by SDS-PAGE with periodic acid-Schiff (PAS) glycan staining [4]. Purified LCC produced in *E. coli* was obtained by a previously described procedure [5].

Scaled-up production of PHL7 was performed in a 300 L bioreactor. 1.0 L preculture medium containing glycerol as the carbon source was inoculated with the *P. pastoris* strain and incubated in shaked flasks. After 24 h at 30°C the preculture was transferred to a Biostat C bioreactor filled up to 10 L with preculture medium. This second preculture was incubated for 24 hours at 30°C keeping the pO_2 at ≥ 30% up to an OD 600_{nm} of 40-50 and was taken as the seed of the 300 L bioreactor filled with 150 L glycerol medium. After a batch phase of 4 hours at 30°C, the glycerol fed-batch phase was started, controlled by a pO_2 of ≥ 30% up to an OD 600_{nm} of ≥ 200. At the following transition phase of 2 hours at pH 6.5, 28°C, 0.2% v/v MeOH was added, while glycerol was decreased linearly at the same time. Then, the methanol

fed-batch was initiated by adding methanol regulated at a pO_2 ≥30%. After 95 h, the fermentation was finished and the enzyme-containing supernatant was separated by centrifugation. Finally the supernatant was concentrated by ultrafiltration and stored at -20°C.

Esterase assay

Esterase activity was quantified with an assay consisting of 0.1 M sodium phosphate pH 7.0 containing 1 mM *p*-nitrophenyl butyrate and 1% v/v isopropanol. The assay was conducted in triplicate in disposable semi-micro cuvettes at 30°C using a Jasco V-730 spectrophotometer equipped with a Peltier PAC-743 cuvette holder. The production rate of *p*-nitrophenol was monitored at 405 nm for 2 to 5 min and converted into enzymatic units (µmol/min) using an extinction coefficient of 9.72 mM^{-1} cm^{-1} [6].

Laboratory-scale enzymatic PET hydrolysis

The degradability of different plastics and effectiveness of polyester hydrolases was assessed by incubating approximately 45 mg PET(-PE) with 25 µg polyester hydrolase in 1 M sodium phosphate buffer (pH 8.0, 1.5 mL) in triplicate at 70°C for 24 hours while shaking at 500 RPM using a ThermoMixer (Eppendorf), unless mentioned otherwise. The residual PET(-PE) was washed with demineralized water, dried and weighed to determine weight loss.

Prior to PET hydrolysis in bioreactors, PET(-PE) was cut into squares of 3x3 cm and milled using an IKA MultiDrive for four times 20 s at 20,000 RPM. The inner temperature of the device did not exceed 50°C during milling. The size of the milled particles was below 16 mm and predominantly 1.25-2.8 mm (41% w/w), followed by 2.8-16 mm (40% w/w) and 0.63-1.25 mm (16% w/w). Crude polyester hydrolase-containing fermentation supernatant was modified to a pH of 8.0 by adding 2 M NaOH and clarified by centrifugation (10,000 *x*g, 4°C) before use. Initial reactions were conducted with a plastic loading of 10 g/L in 3.6 L Labfors bioreactors (Infors, Bottmingen, Switzerland) with a stirrer, baffles, temperature sensor and pH electrode. The starting liquid volume was 1.0 L. Bioreactors were loaded with MilliQ water or buffer solution, set to temperature, crude polyester hydrolase was added, and the reaction started by adding 10 g of PET(-PE). Reactions were stirred at 250 RPM using a marine impeller. The pH was maintained at 8.0 by addition of 2 M NaOH. Subsequent reactions at higher plastic loadings were conducted in 0.5 L Multifors bioreactors (Infors). Methodology was the same, except for an increased stirring speed of 500 RPM. Reactions with plastic loadings of 12.5-25 g/L were conducted with a starting liquid volume of 200-400 mL and 5.0 g plastic. Reactions with plastic loadings of 100-200 g/L were conducted with a total starting volume of 200 mL and 20.0-40.0 g plastic. The volume of 2 M NaOH added was determined gravimetrically using an experimentally determined density of 1.08 g/mL. Samples were taken at regular time intervals using and stored at 4°C until analysis.

Pilot-scale enzymatic PET hydrolysis

Pilot-scale hydrolysis of PET embedded within 4.5 kg of PET-PE multilayer material was performed in a 120 L reactor (Órbita Ingeniería, Valencia, Spain), allowing control over stirring, temperature and pH (Fig. S2). First, the reactor's jacket temperature was established, then demineralized water and salts were added to achieve concentrations of 1 M NaCl and 0.2 M sodium phosphate. Subsequently, PET-PE flakes were introduced into the reactor. The pH was adjusted to 8.0 by adding 12 M NaOH, and the reactor temperature was maintained at 65°C. Once both the temperature and pH were stable, the reaction was

started by adding enzyme. The total reaction volume was 90 L, and the reaction mixture was stirred at 375 RPM using a crossed blade impeller. The reactions were allowed to proceed for 144 h.

Downstream processing

Upon completion of a hydrolysis reaction, residual solids were collected by filtration, washed with demineralized water, dried at 60°C overnight, and weighed. The percentage of PET depolymerized (*Y*) was calculated as follows:

$$Y = \frac{m_{end} - m_{PE}}{m_{start} - m_{PE}} \cdot 100\%$$

with m_{PE} denoting the mass quantity of PE in the PET-PE multilayer material, calculated by subtracting the PET mass fraction quantified by GPC from the total PET-PE mass quantity. It was assumed that the PE mass did not change during PET hydrolysis. The reaction liquid was further clarified by centrifugation or filtration over a paper filter. TPA was precipitated through addition of 1 M sulfuric acid, under stirring, until the pH reached 2.0, after which the suspension was filtered over a P3 sintered glass filter, the residue washed with demineralized water, and dried in a vacuum oven at 40°C for 48 h. Prior to analyses, the dry TPA was ground to a powder.

To obtain high purity PE from the residual solids fraction obtained from the reaction mixture, it was treated by alkaline hydrolysis. Solids were suspended in 2.5 M NaOH containing 12.5 g/L methyltrioctylammonium bromide, and incubated for 16 h at 95°C while softly shaking. The cleaned PE was obtained by filtration, washed with demineralized water and acetone, and dried at 105°C.

To evaluate the processability of recovered PE, a lab-scale twin-screw extruder MC 15 (Xplore Instruments, Sittard, The Netherlands) was used at 100 RPM screw rotation speed. It was coupled to a winding system to obtain PE films (Fig. S3). Crude residual solids were processed at 260-270°C with a residence time of 3 min, while PE cleaned by alkaline hydrolysis was processed at 190-200°C with a residence time of 4 min.

Repolymerization of terephthalic acid

TPA (2.5 g, 15.0 mmol), ethylene glycol (10 mL, 179.4 mmol) and germanium(IV) oxide (4 mg, 38.2 µmol) were charged into a 100 mL 3-neck round bottom flask. The flask was equipped with a PTFE overhead stirrer and a distillation setup consisting of a 3-way distillation head, a receiver adapter with nitrogen/vacuum inlet and a receiving flask. The setup was evacuated and refilled with nitrogen three times. The reaction mixture was stirred and heated on a hot plate. During 30 min, the temperature was gradually increased to 200°C (externally measured) and the reaction mixture was kept at this temperature for 4.5 h. At this point, all solids had dissolved and the temperature was further increased to 265 °C during 30 min, while distilling off the excess ethylene glycol. Then, a vacuum reaching 0.01 mbar was gradually applied and the polymerization reaction was continued for an additional 2.5 h under the vacuum. The mixture was allowed to cool down to room temperature under nitrogen, yielding the PET polymer as an opaque solid.

Analytical methods

For analysis of PET(-PE), gel-permeation chromatography (GPC), differential scanning calorimetry (DSC) and Fourier transform infrared spectroscopy (FTIR) were performed as described previously [7]. For GPC of PET, PET from PET-PE was dissolved in hexafluoroisopropanol, which does not dissolve PE. PTA-PET Invista RT4048 was used as reference PET for calculating concentrations, dn/dc values, and molecular weight distributions. For analysis of recovered PE, DSC measurements were performed using a DSC Q2000 (TA Instruments, New Castle, US) under 50 mL/min N_2 gas, by first pre-heating the sample to 300°C, then cooling the sample to -50°C, and again heating the sample to 300°C (10°C/min). The PE density was measured according to ISO 1183-1. A melt-flow index (MFI) analysis to determine the rheological properties of PE was carried out according to ISO1133-1 on a MI-3 melt flow indexer (GÖTTFERT, Buchen, Germany), using a pre-heating time of 300 s, a loading weight of 2.16 kg and a temperature of 190 ºC. For the crude residual solids, it was necessary to increase the loading weight to 21.6 kg.

Tensile tests on PE films were performed according to ISO 527 on a Universal testing machine M350-20CT (Testometric; Rochdale, UK). The samples were conditioned at 23°C and 50% humidity for at least 24 h. At least five samples were tested per measurement. Diffuse opacity and color was measured with a CM-3600D spectrophotometer (Konica Minolta, Tokyo, Japan). Opacity here represents the ratio of the diffuse light reflectance factor of a film with a black background, expressed as percentage. Color was measured according to CIELAB coordinates of lightness, red-green and yellow-blue.

Aromatic PET monomer concentrations were quantified by reverse-phase high-performance liquid chromatography (RP-HPLC). Liquid samples were first heated to 95°C for 10 min to inactivate enzyme and filtered over a 0.2 µm pore size regenerated cellulose filter (Whatman, Maidstone, UK). Solid samples were dissolved in 0.25 M Tris buffer (pH 8.0). TPA and isophthalic acid standards were dissolved in 0.1 M NaOH. Mono(2-hydroxyethyl) terephthalic acid (MHET) and bis(2-hydroxyethyl) terephthalate (BHET) standards were dissolved in methanol. Analyte separation was performed with a Waters Alliance HPLC (Millford, MA, US) on an XBridge BEH C18 column (5.0 µm, 4.6x250 mm) at 30°C and a flow of 0.8 mL/min. Eluent consisted of 70% v/v MilliQ water containing 3 mM sulfuric acid and 30% v/v methanol. Analytes were detected using a 2489 UV/Vis detector (Waters) at 240 nm.

For proton nucleic magnetic resonance spectroscopy (^{1}H-NMR), spectra were recorded at 298 K on a Bruker Avance III spectrometer (400.17 MHz). TPA samples were dissolved in deuterated dimethyl sulfoxide. PET samples were dissolved in a 6:1 (v/v) mixture of deuterated chloroform and trifluoroacetic acid. Maleic acid was used as an internal standard to quantify the purity of TPA recovered after the depolymerization reaction.

References

(1) Shirke, A. N.; White, C.; Englaender, J. A.; Zwarycz, A.; Butterfoss, G. L.; Linhardt, R. J.; Gross, R. A. Stabilizing Leaf and Branch Compost Cutinase (LCC) with Glycosylation: Mechanism and Effect on PET Hydrolysis. *Biochemistry* **2018**, *57* (7), 1190-1200. DOI: 10.1021/acs.biochem.7b01189.
(2) Zhang, W.; Bevins, M. A.; Plantz, B. A.; Smith, L. A.; Meagher, M. M. Modeling *Pichia pastoris* growth on methanol and optimizing the production of a recombinant protein, the heavy-chain fragment C of botulinum neurotoxin, serotype A. *Biotechnol Bioeng* **2000**, *70* (1), 1-8. DOI: 10.1002/1097-0290(20001005)70:1<1::aid-bit1>3.0.co;2-y.

(3) Werten, M. W. T.; Moers, A. P. H. A.; Vong, T.; Zuilhof, H.; van Hest, J. C. M.; de Wolf, F. A. Biosynthesis of an Amphiphilic Silk-Like Polymer. *Biomacromolecules* **2008**, *9* (7), 1705-1711. DOI: 10.1021/bm701111z.
(4) Zacharius, R. M.; Zell, T. E.; Morrison, J. H.; Woodlock, J. J. Glycoprotein staining following electrophoresis on acrylamide gels. *Analytical Biochemistry* **1969**, *30* (1), 148-152. DOI: https://doi.org/10.1016/0003-2697(69)90383-2.
(5) Sonnendecker, C.; Oeser, J.; Richter, P. K.; Hille, P.; Zhao, Z.; Fischer, C.; Lippold, H.; Blazquez-Sanchez, P.; Engelberger, F.; Ramirez-Sarmiento, C. A.; et al. Low Carbon Footprint Recycling of Post-Consumer PET Plastic with a Metagenomic Polyester Hydrolase. *ChemSusChem* **2022**, *15* (9), e202101062. DOI: 10.1002/cssc.202101062.
(6) Gamerith, C.; Zartl, B.; Pellis, A.; Guillamot, F.; Marty, A.; Acero, E. H.; Guebitz, G. M. Enzymatic recovery of polyester building blocks from polymer blends. *Process Biochemistry* **2017**, *59*, 58-64. DOI: 10.1016/j.procbio.2017.01.004.
(7) Maaskant, E.; van Es, D. S. Unexpected Susceptibility of Poly(ethylene furanoate) to UV Irradiation: A Warning Light for Furandicarboxylic Acid? *ACS Macro Letters* **2021**, *10* (12), 1616-1621. DOI: 10.1021/acsmacrolett.1c00676.

Supplementary Figures and Tables

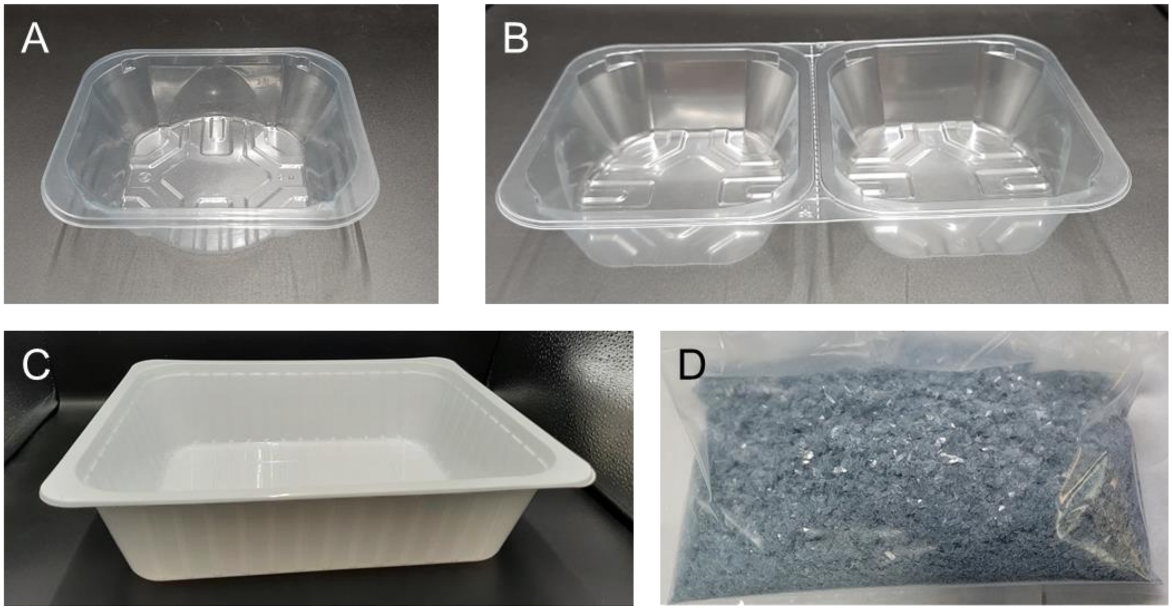

Figure S1. PET-PE materials used in this study. A: PET-PE#1. B: PET-PE#2. C: PET-PE#3. D: PET-PE production waste (4.5 kg).

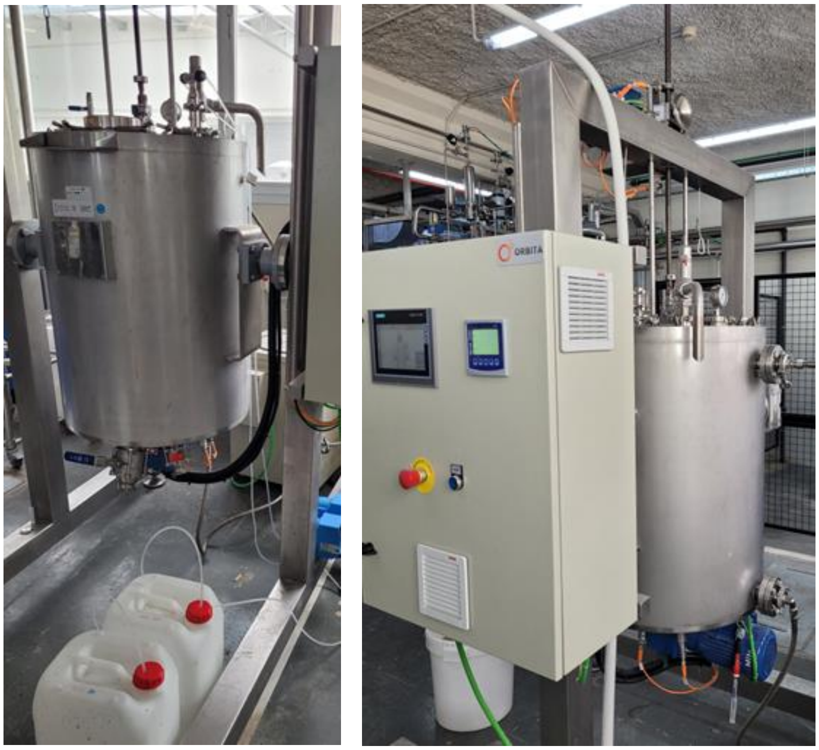

Figure S2: 120 L scale reactor used in this study.

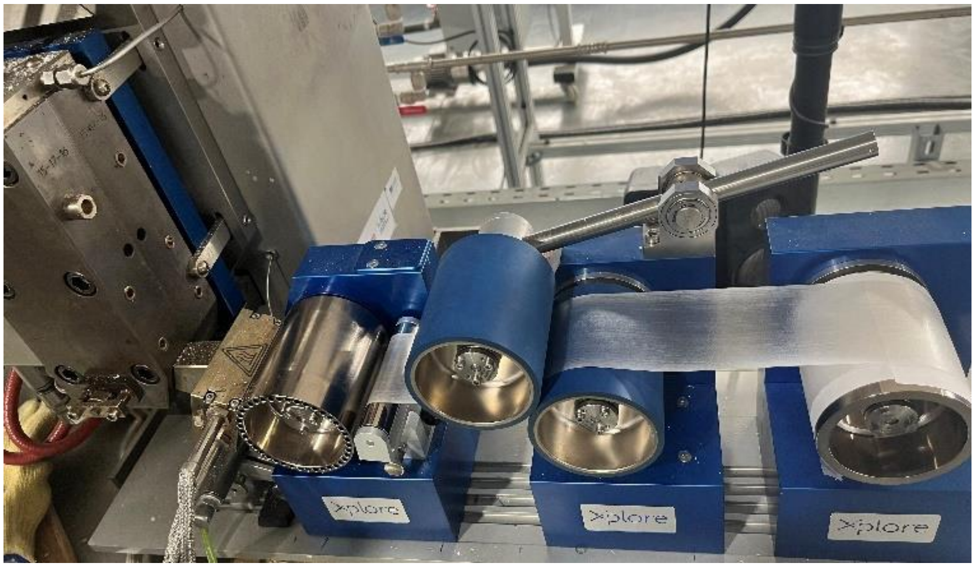

Figure S3: the winding system used for PE processing.

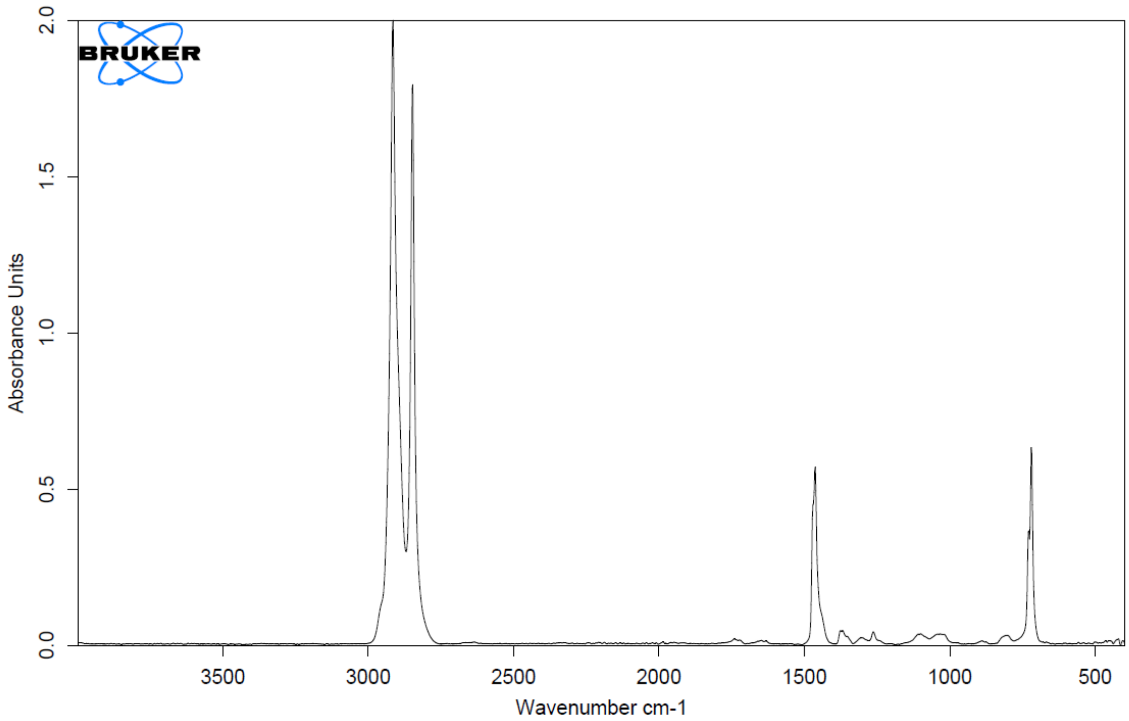

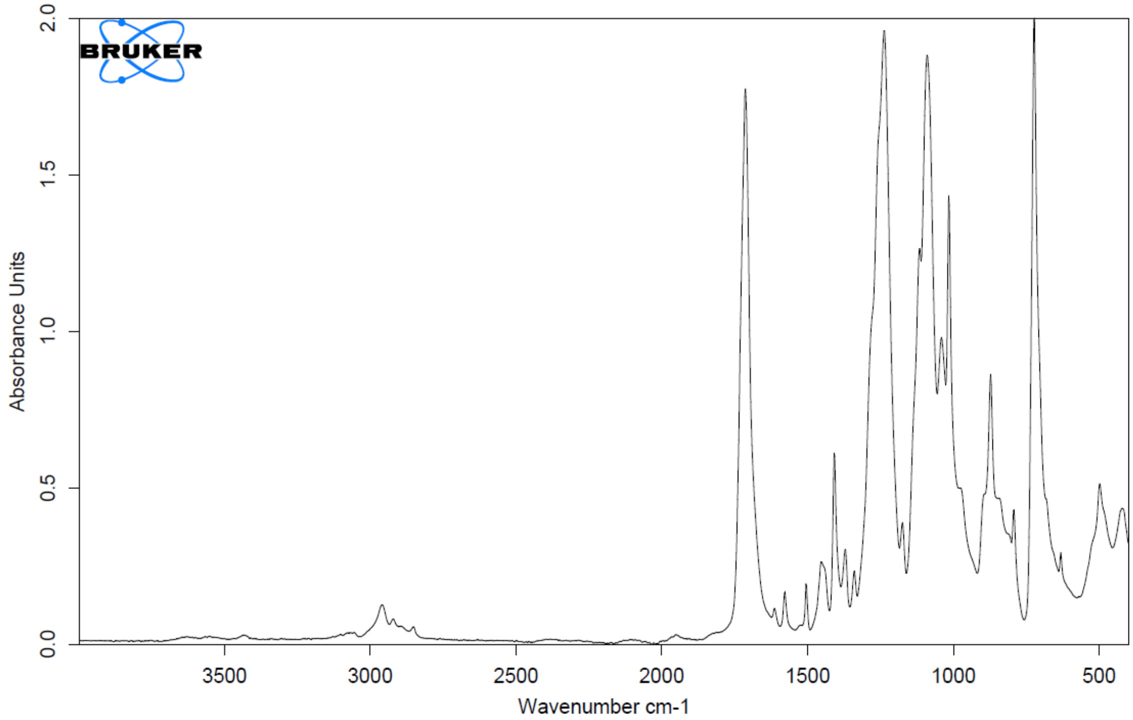

Figure S4: FTIR spectra of PET-PE#1. Top is the spectrum of the PE layer, bottom of the PET layer.

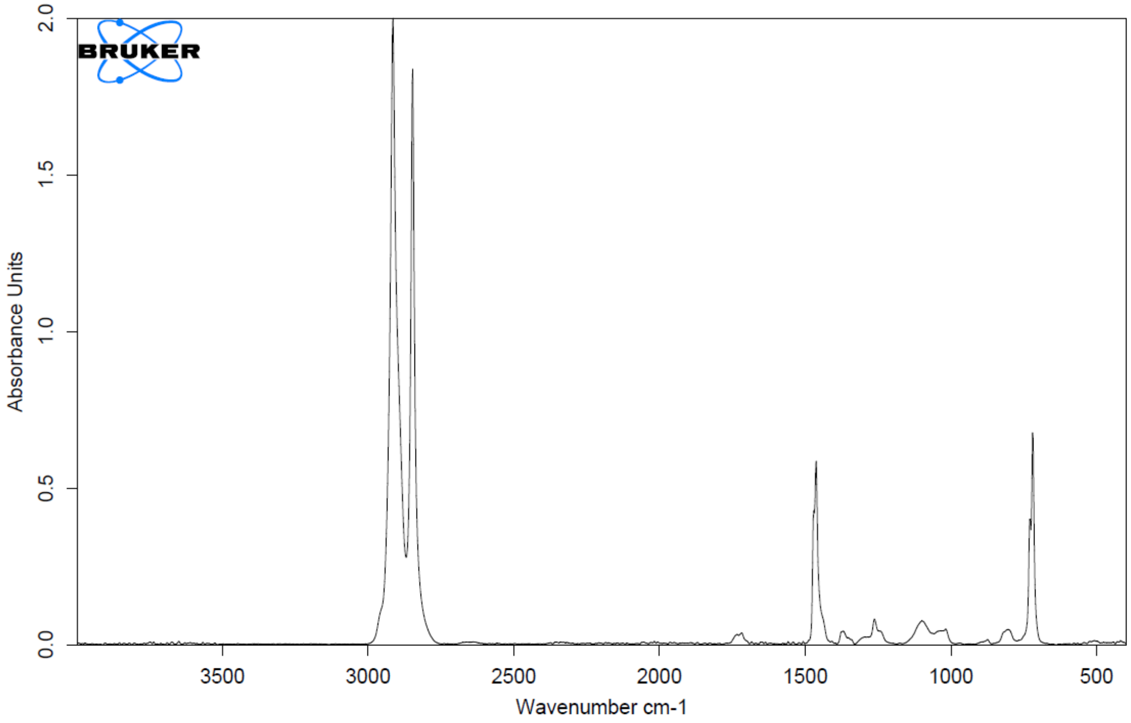

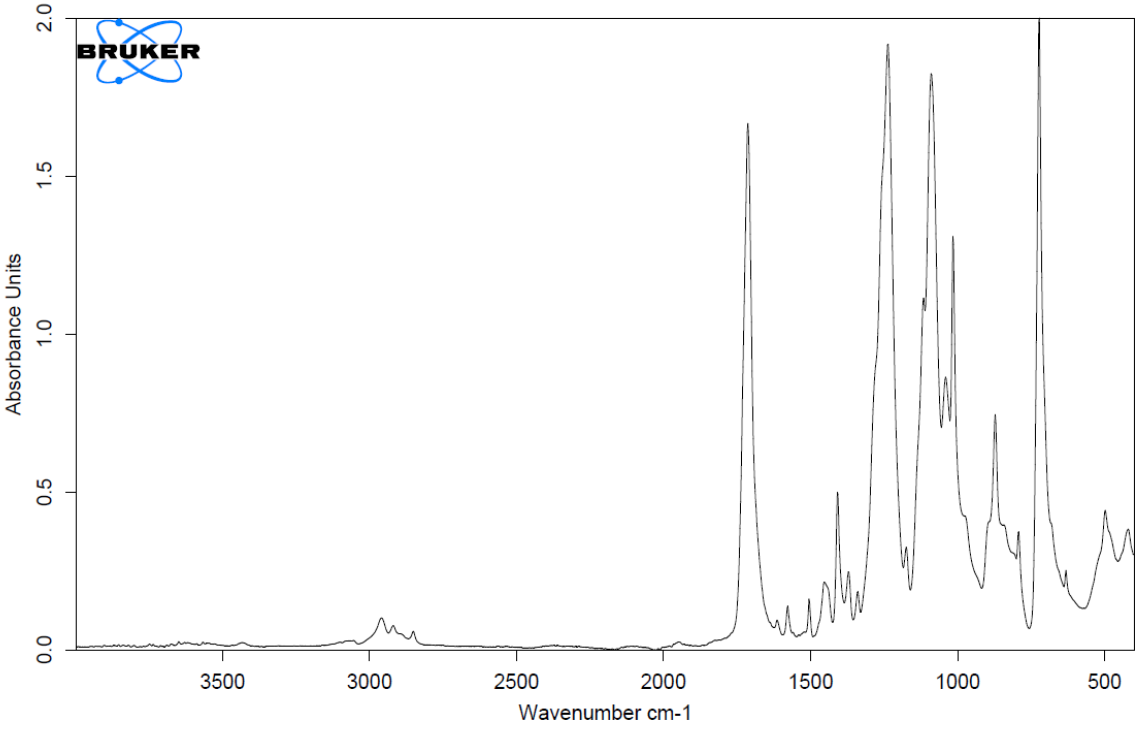

Figure S5: FTIR spectra of PET-PE#2. Top is the spectrum of the PE layer, bottom of the PET layer.

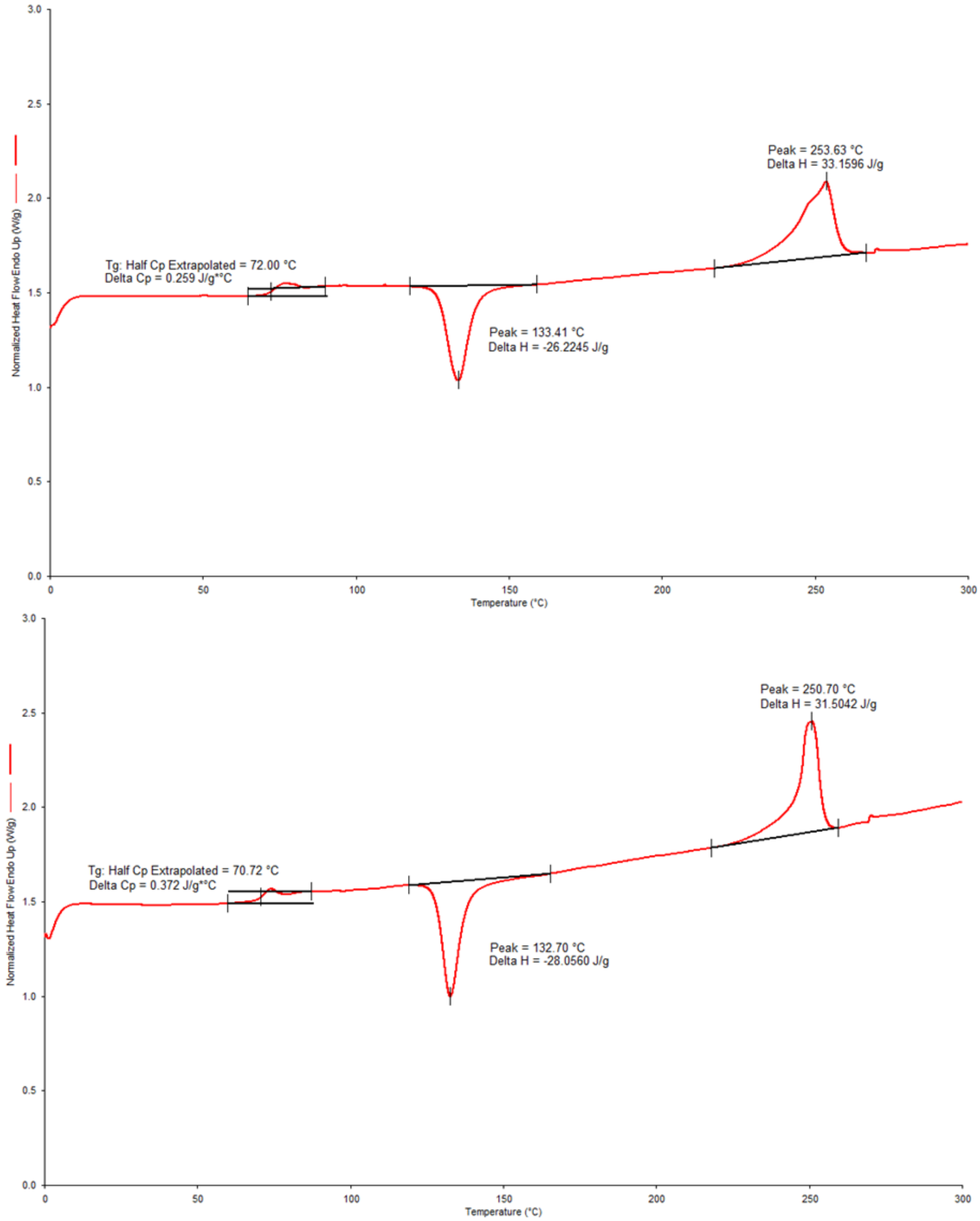

Figure S6: DSC analysis of amorphous PET sheet from Goodfellow (Gf-PET) as obtained from the supplier (top) and after milling to reduce particle size (bottom).

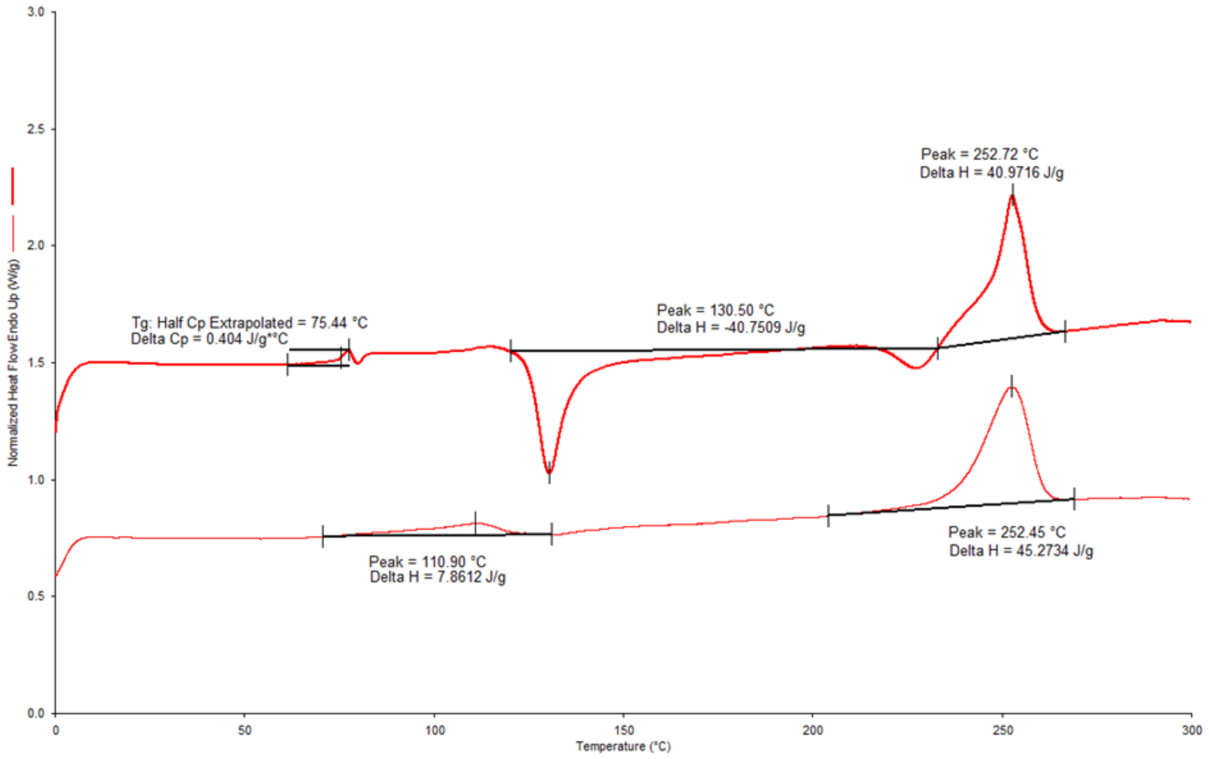

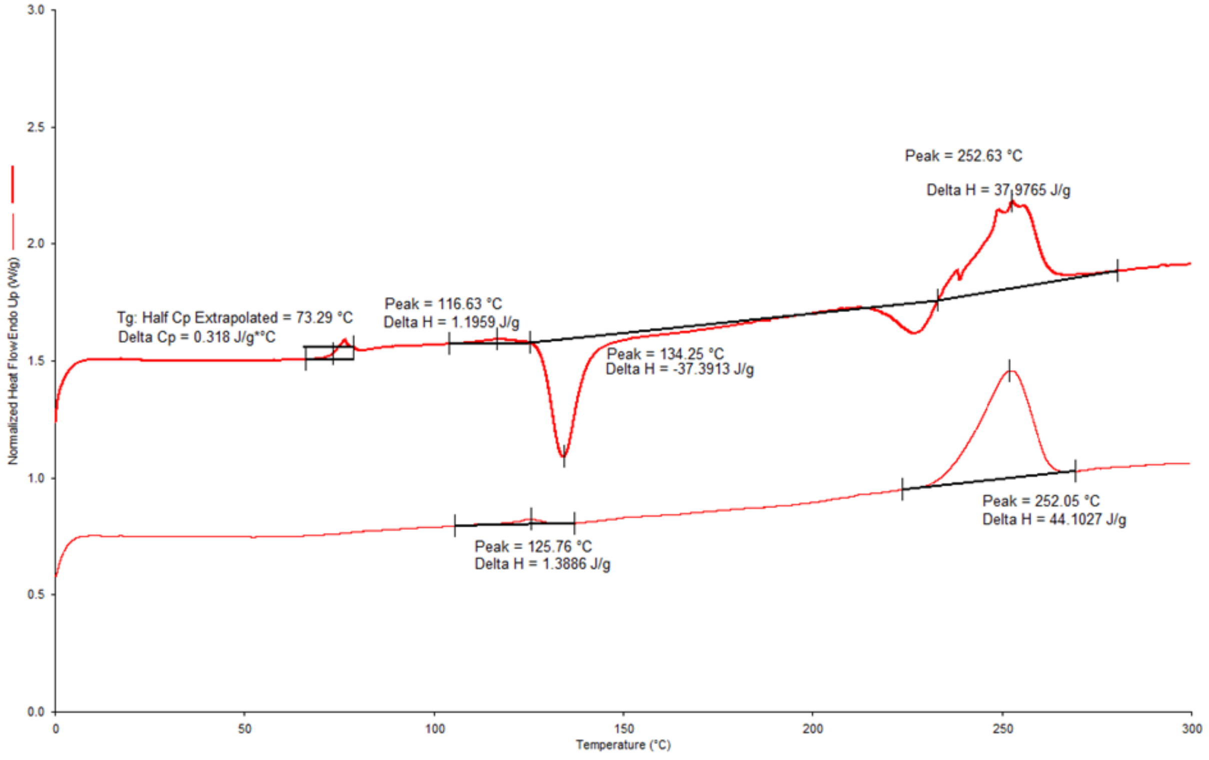

Figure S7: DSC analysis of PET-PE#1 (top) and PET-PE#2 (bottom). In both graphs, the bold top trace shows the first heating cycle, showing a (negative) crystallisation peak at around 130°C. The thinner bottom trace shows the second heating cycle, in which the PET has already been fully crystallised by the first cycle.

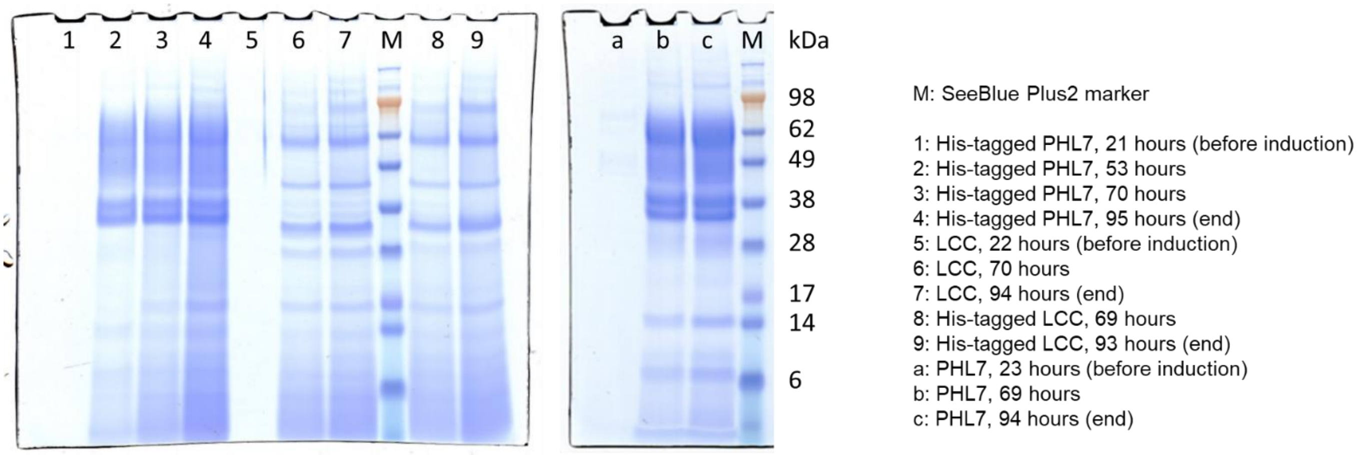

Figure S8: SDS-PAGE analysis of the culture supernatant of *Pichia pastoris* overexpressing polyester hydrolases. The loading volume was 15 µL for samples and 10 µL for the marker.

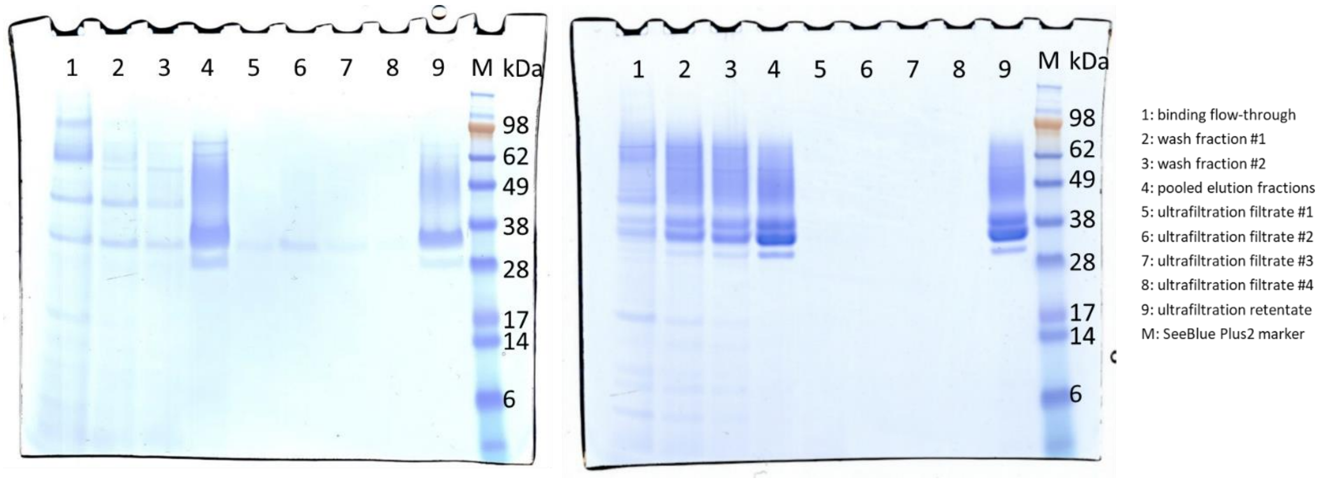

Figure S9: SDS-PAGE analysis of the progress of IMAC purification of His-tagged LCC (left gel) and PHL7 (right gel). The loading volume was 15 µL for lanes 1 to 3, 7.5 µL for lanes 4 to 9, and 10 µL for the marker.

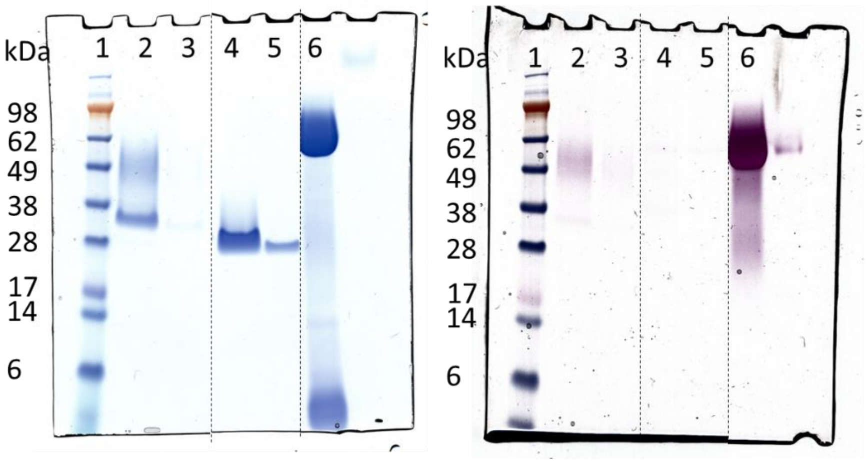

1. SeeBlue Plus2 marker (pre-stained)
2. Purified His-tagged LCC produced in *Pichia pastoris*
3. Purified His-tagged LCC produced in *Pichia pastoris*, diluted five-fold
4. Purified His-tagged LCC produced in *E. coli* (negative control for glycosylation)
5. Purified His-tagged LCC produced in *E. coli*, diluted five-fold (negative control for glycosylation)
6. Purified non-related glycosylated protein produced in *Pichia pastoris*, used as positive control for glycosylation

Figure S10: SDS-PAGE analysis of purified LCC expressed in *Pichia pastoris* (1.5 µg) and *Escherichia coli* (3.4 µg). Left gel: Coomassie Blue staining, Right gel: PAS staining to visualize glycosylation. Dashed lines indicate where irrelevant parts of the gel pictures were removed for clarity of presentation.

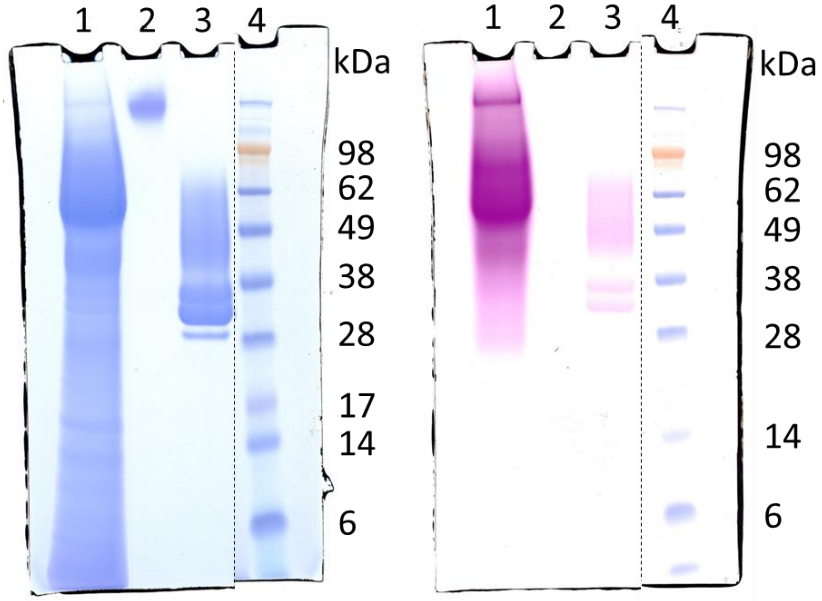

1. Purified non-related glycosylated protein produced in *Pichia pastoris*, used as positive control for glycosylation
2. Purified non-related non-glycosylated protein produced in *Pichia pastoris* (negative control, 15 µg)
3. purified His-tagged PHL7 produced in *Pichia pastoris* (19 µg)
4. SeeBlue Plus2 marker (pre-stained)

Figure S11: SDS-PAGE of purified PHL7 expressed in *Pichia pastoris*. Left gel: Coomassie Blue staining. Right gel: PAS staining to visualize glycosylation. Dashed lines indicate where irrelevant parts of the gel pictures were removed for clarity of presentation.

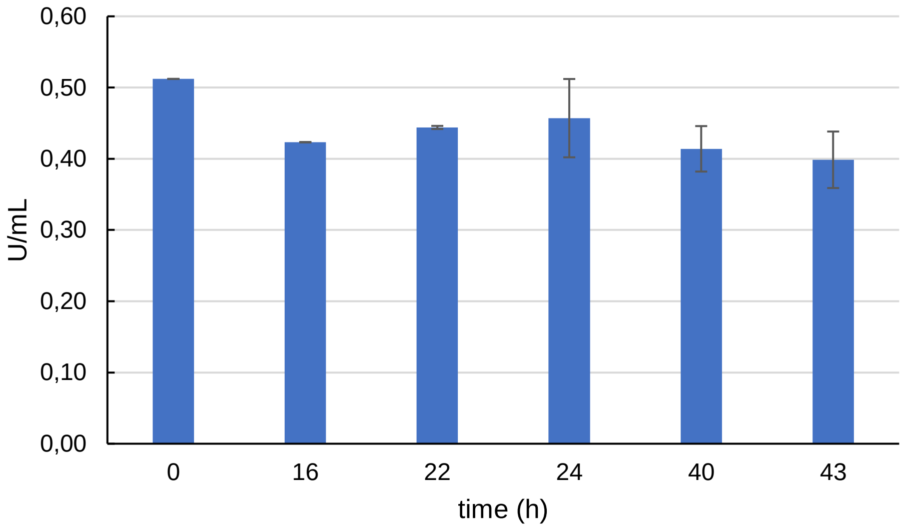

Figure S12: Esterase activity in solution during enzymatic PET hydrolysis of 10 g Gf-PET using crude LCC in bioreactors, at 10 g_{PET}/L, 0.8 mg_{LCC}/g_{PET} (75 U/g_{PET}), 70°C, and pH 8.

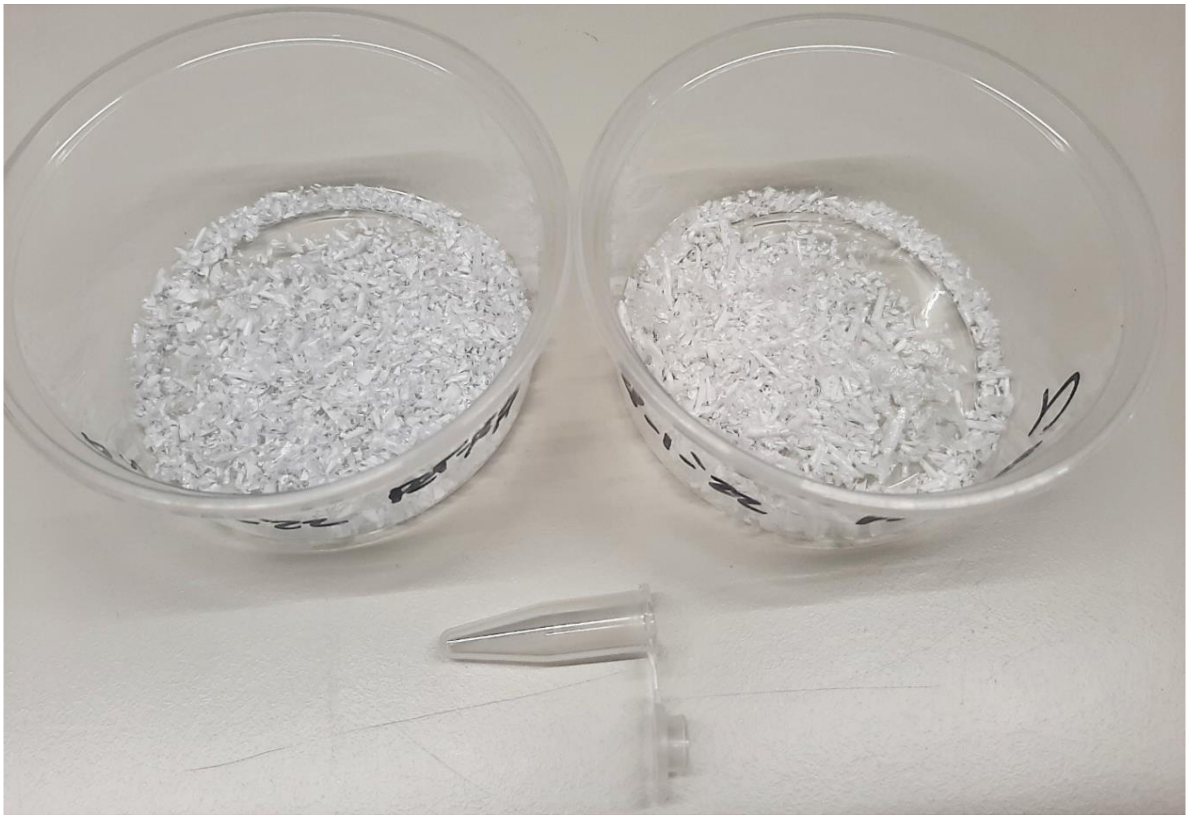

Figure S13: Residual solids isolated from enzymatic hydrolysis of milled PET-PE packaging at 70 °C. 1.5 mL Eppendorf tube shown for scale. Left: PET-PE#1, Right: PET-PE#2.

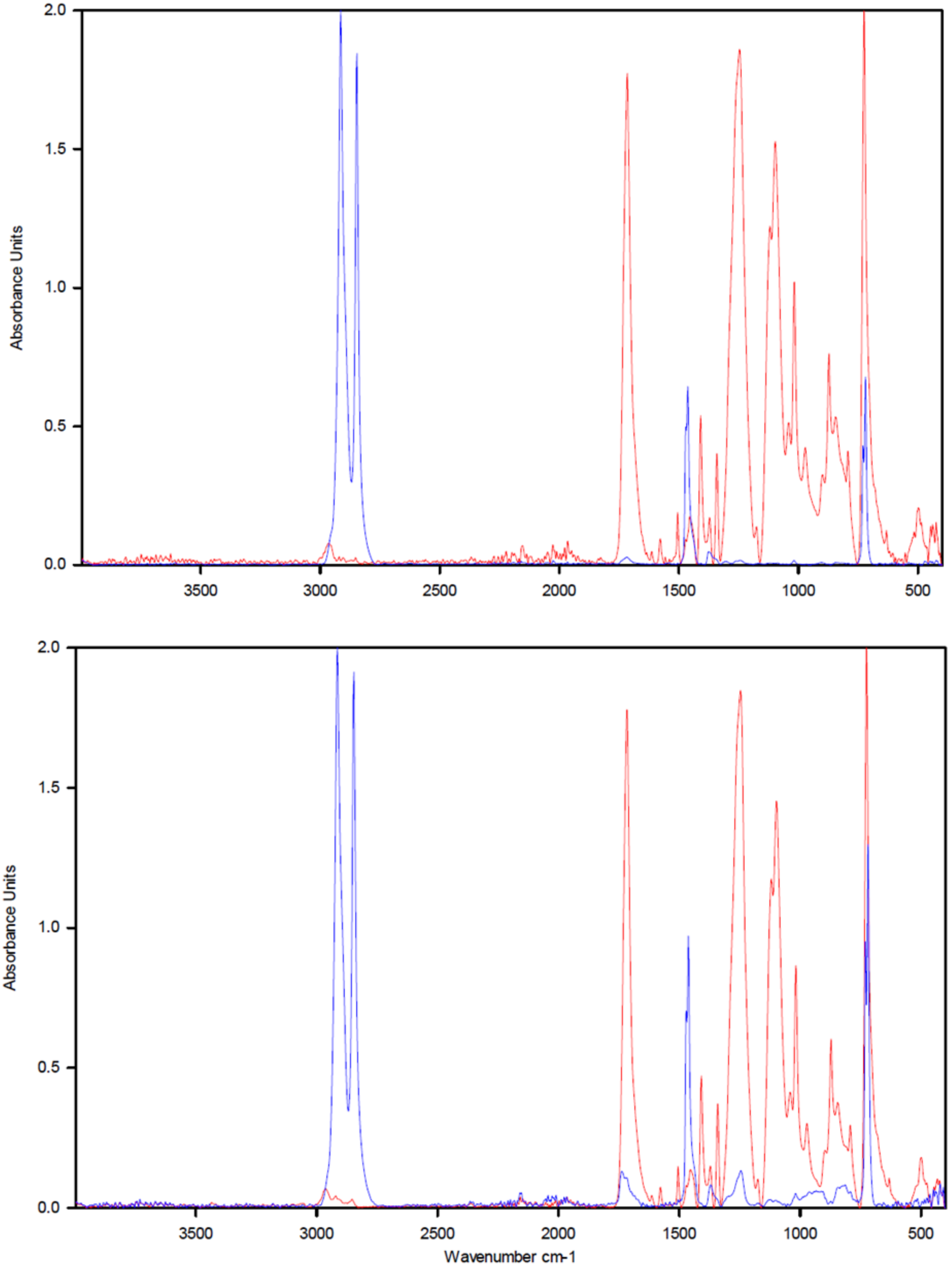

Figure S14: FTIR analysis of PET (red spectra) and PE (blue spectra) sides of residual PET-PE#1 (top) and PET-PE#2 (bottom) after enzymatic hydrolysis at 70 °C.

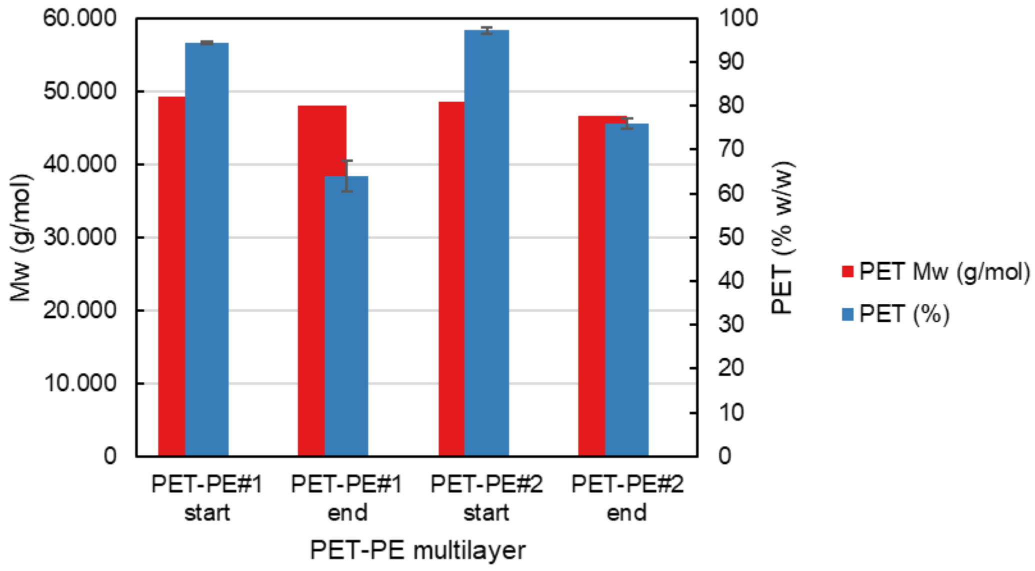

Figure S15: GPC analysis of starting and residual PET-PE#1 and PET-PE#2 isolated from enzymatic hydrolysis reactions at 70 °C.

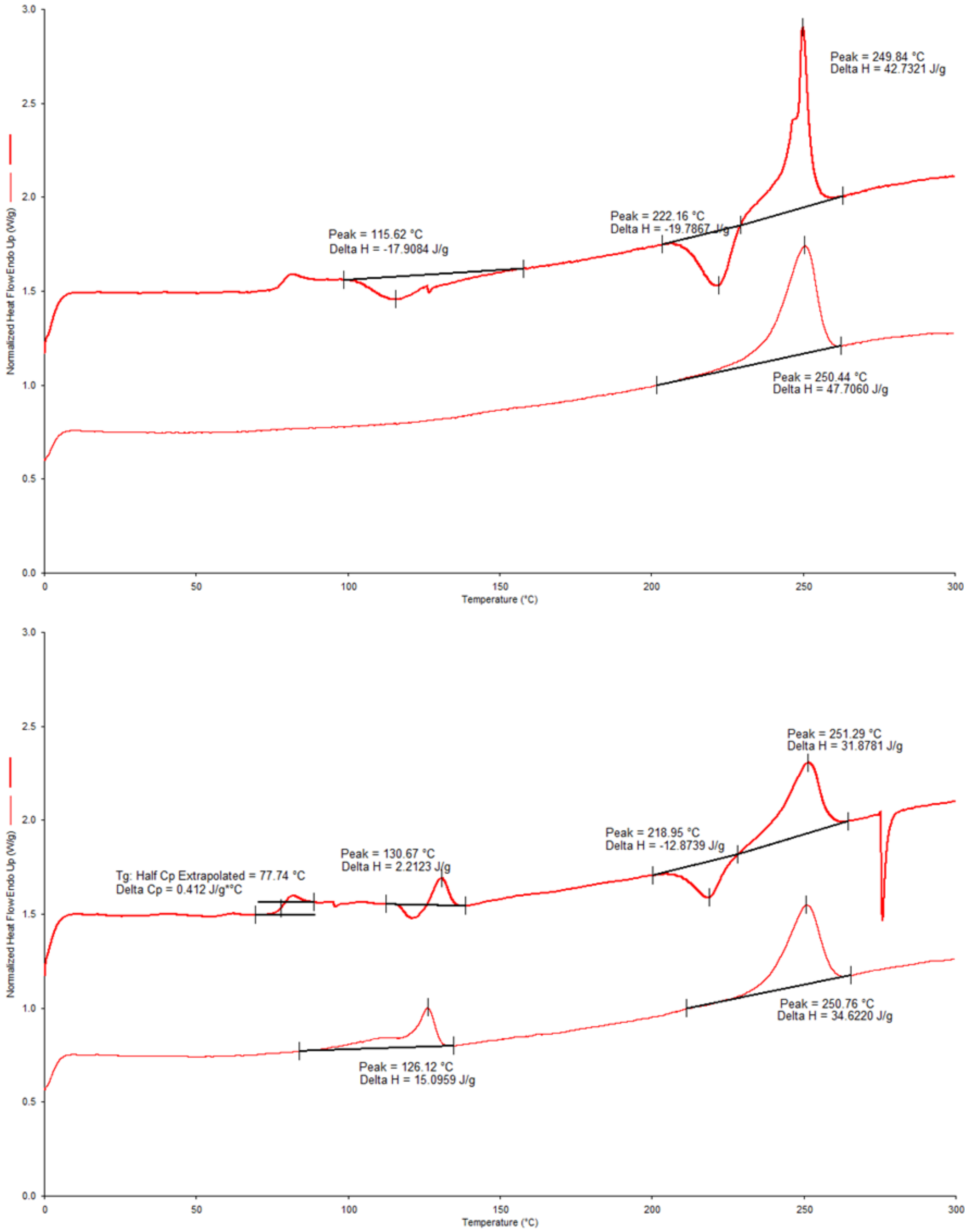

Figure S16: DSC analysis of residual PET-PE#1 (top) and PET-PE#2 (bottom) isolated from enzymatic hydrolysis reactions at 70 °C. In both graphs, the bold top trace shows the first heating cycle. The thinner bottom trace shows the second heating cycle, in which the PET has already been fully crystallised by the first cycle.

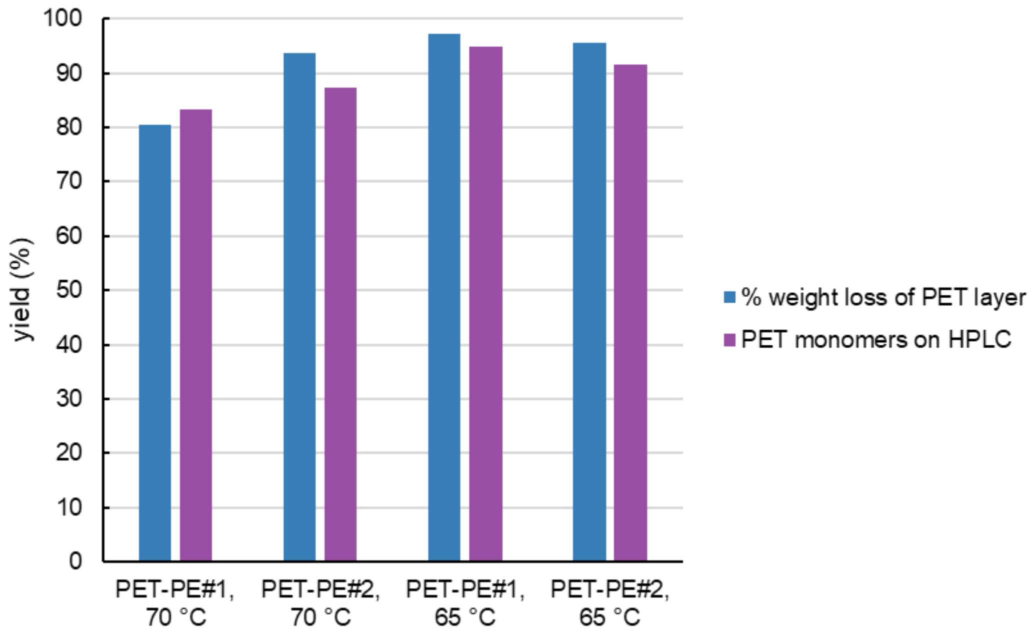

Figure S17: Extent of depolymerisation of PET in PET-PE#1 and PET-PE#2 after enzymatic hydrolysis reactions performed for 115 h at different temperatures, determined from the weight loss of solids and determination of the concentration of aromatic PET monomers by HPLC.

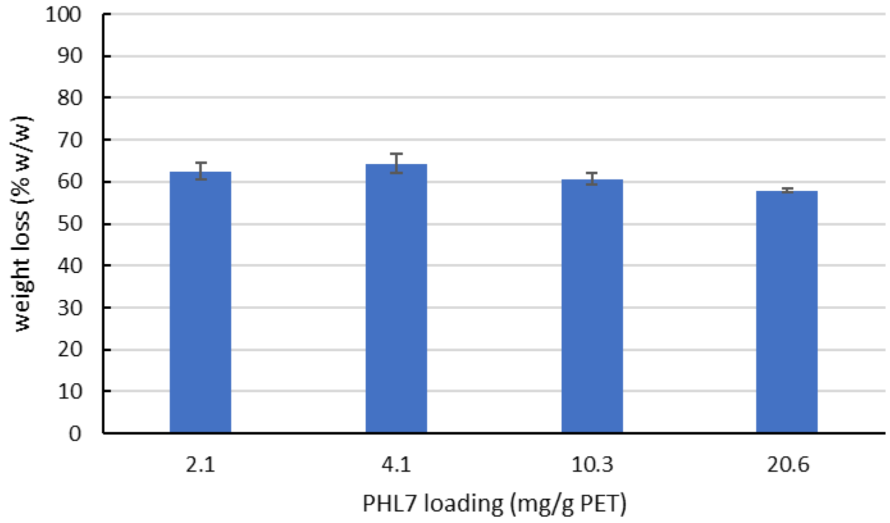

Figure S18: Effect of different loadings of crude PHL7 (culture supernatant from *Pichia pastoris* fermentation) on the degradation of Gf-PET in small scale hydrolysis assays. Assays were performed as described in section 2.4 of the main manuscript, except for the variation in the enzyme loading.

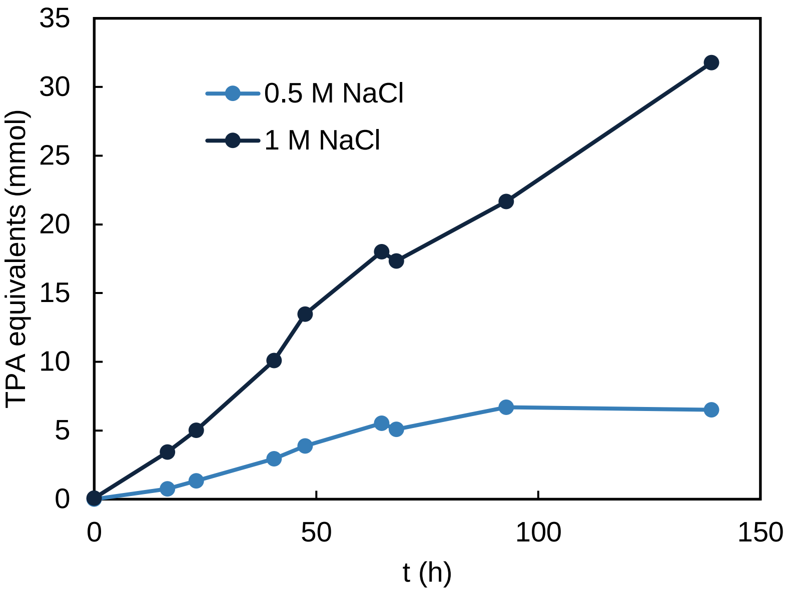

Figure S19. Accumulation of dissolved aromatic monomers over time resulting from enzymatic PET hydrolysis of 5.0 g PET-PE#2 with crude PHL7 in bioreactors, at 25 $g_{PET\text{-}PE}$/L, 100 U/$g_{PET\text{-}PE}$ (4 mg_{PHL7}/$g_{PET\text{-}PE}$), 65°C, and pH 8 in the presence of 0.5 M or 1 M NaCl. TPA equivalents concentration consists of TPA, MHET and isophthalic acid concentrations summed.

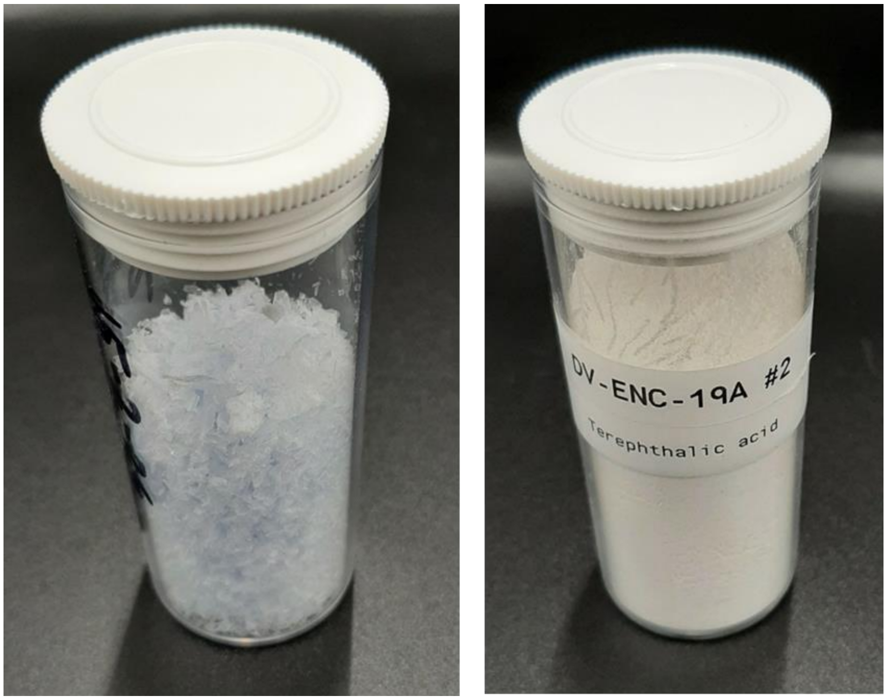

Figure S20. Residual plastic remaining after an enzymatic hydrolysis reaction with 200 g/L PET-PE#2 (left), and terephthalic acid isolated from the hydrolysate (right).

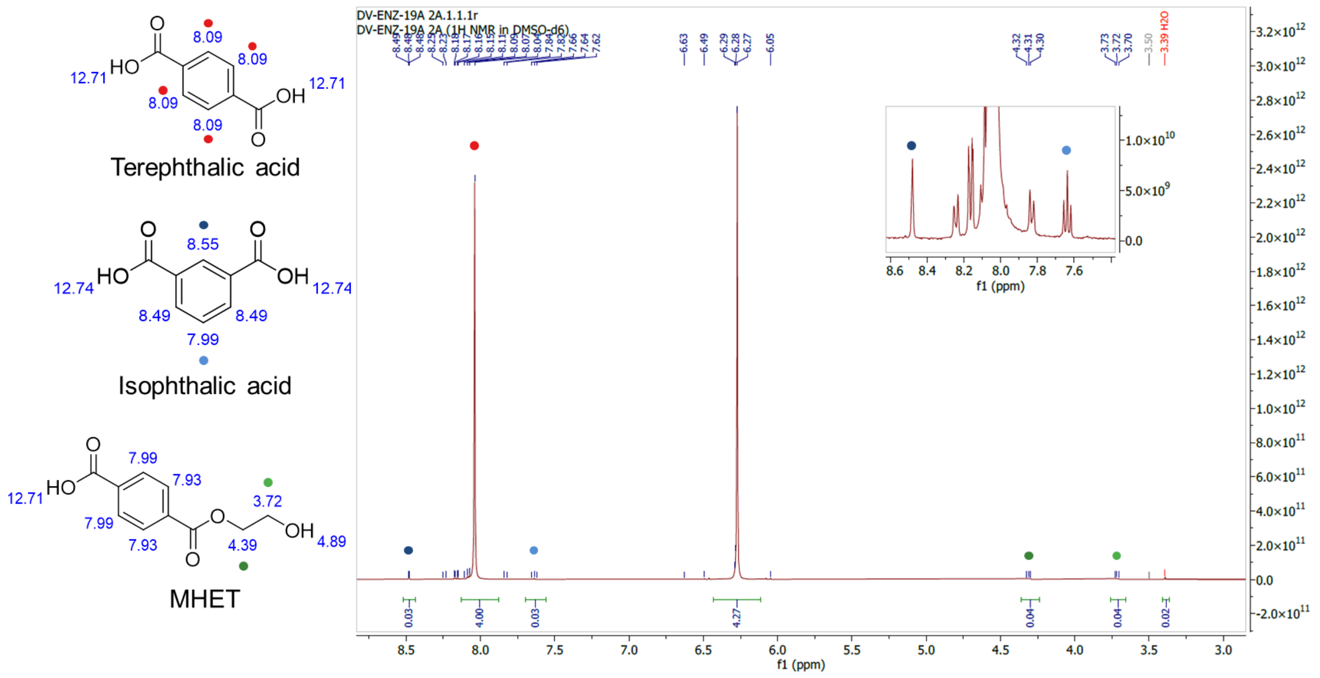

Figure S21. Annotated ^{1}H-NMR spectrum of terephthalic acid recovered from laboratory-scale PET-PE hydrolysate of 200 g/L PET-PE#2. Chemical structures on the left include the proton chemical shifts predicted using ChemDraw 18.0 in blue.

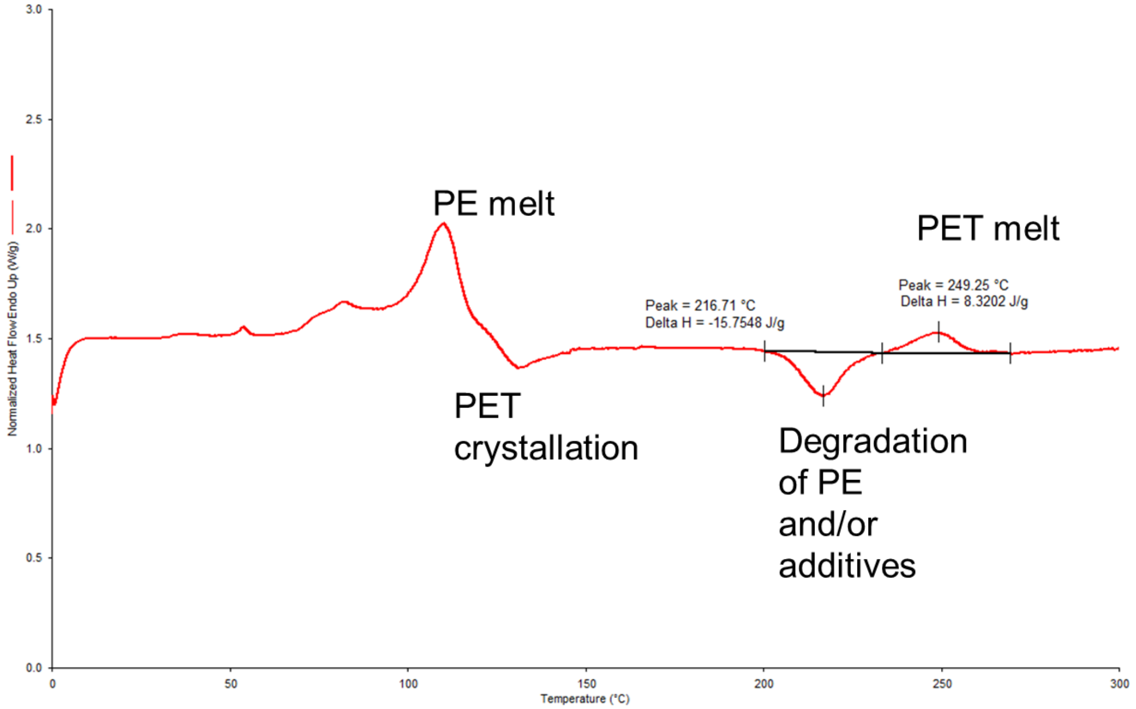

Figure S22: DSC analysis of residual plastic after enzymatic hydrolysis reaction employing crude PHL7 with 200 g/L PET-PE#2 as substrate.

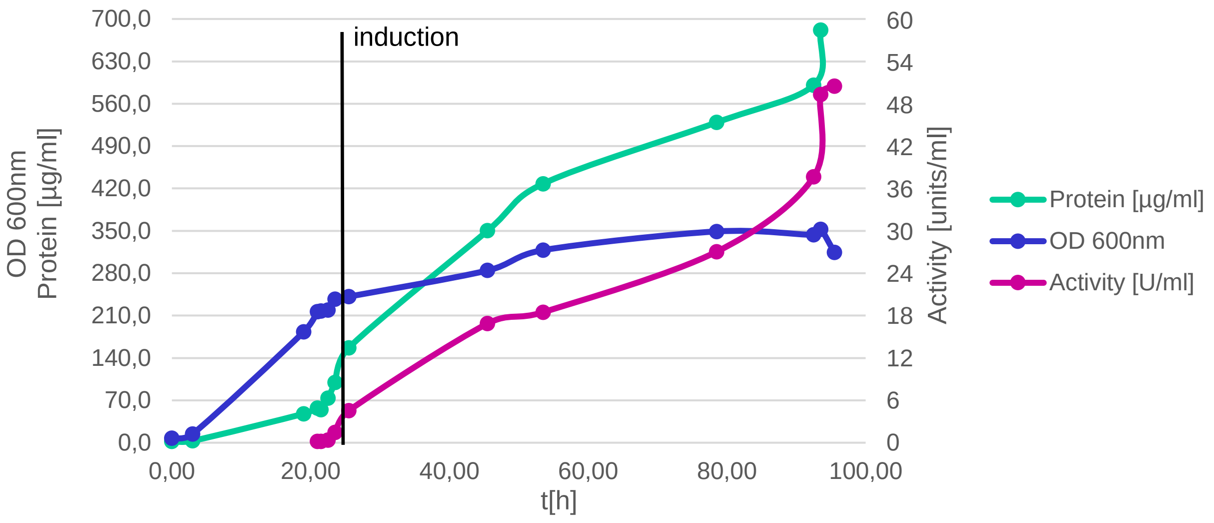

Figure S23. Scale up of the fermentation process for production of PHL7 in *Pichia pastoris* in a 300 L bioreactor. Enzymatic activity, protein concentration and cell density are shown in dependency of cultivation time.

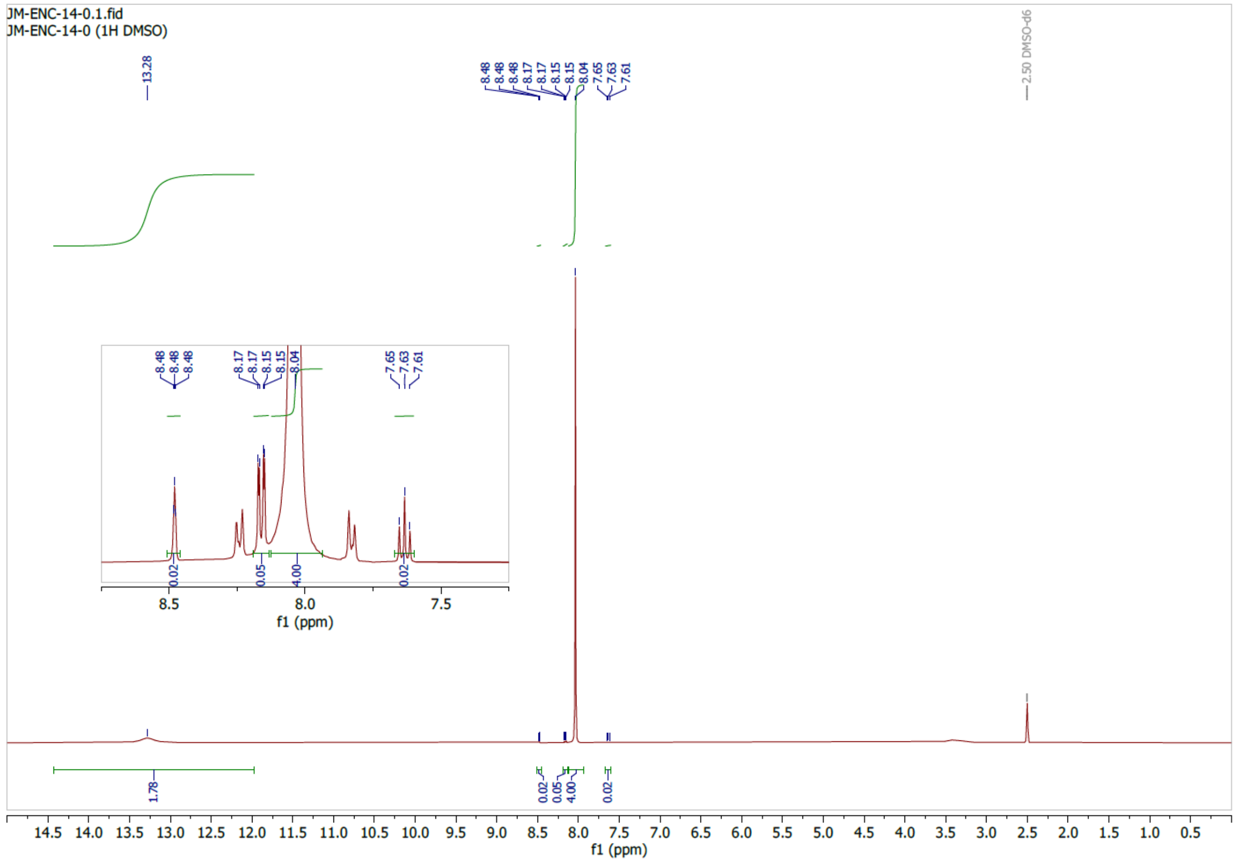

Figure S24: ^{1}H-NMR spectrum of crude terephthalic acid recovered from pilot scale enzymatic hydrolysis reactions using waste from PET-PE production as substrate.

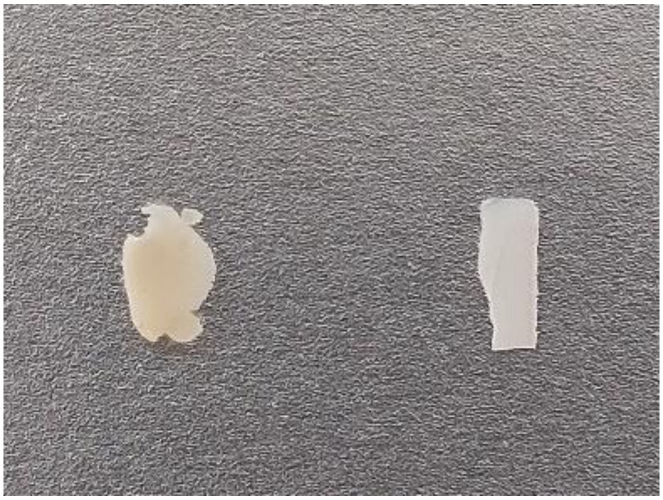

Figure S25. Visual appearance of the polymers obtained from the repolymerization of crude terephthalic acid isolated from enzymatic hydrolysis reactions (left) and terephthalic acid washed with hot ethylene glycol prior to repolymerization (right).

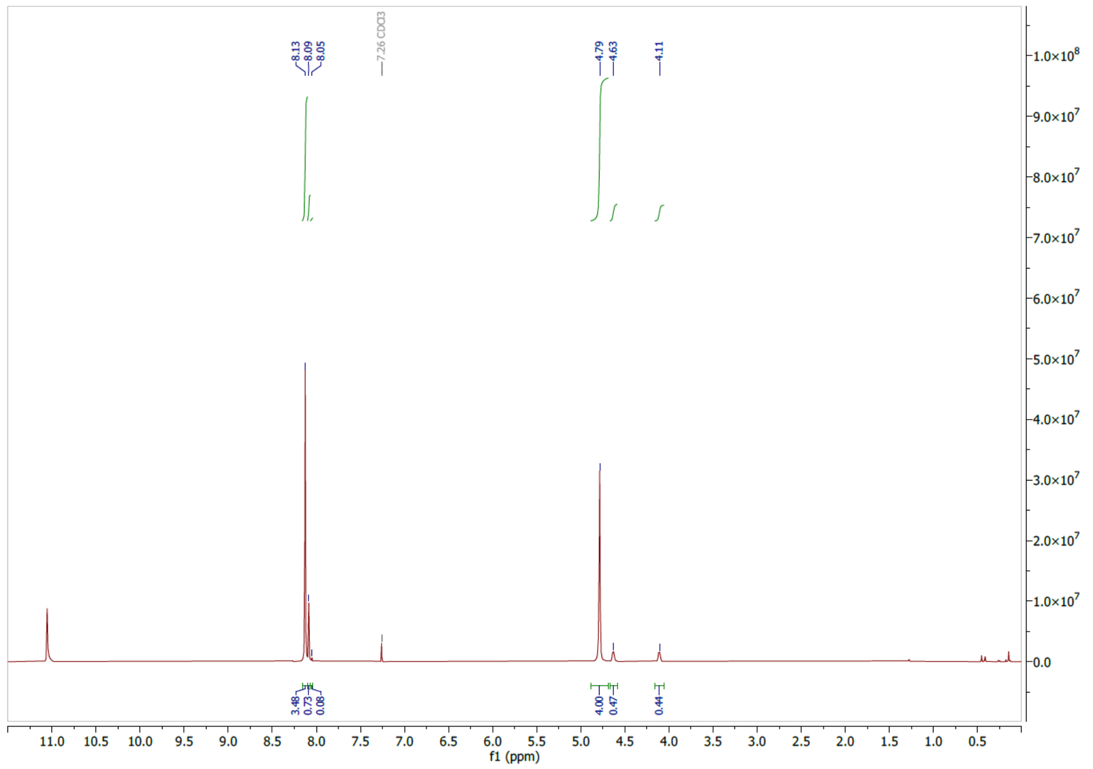

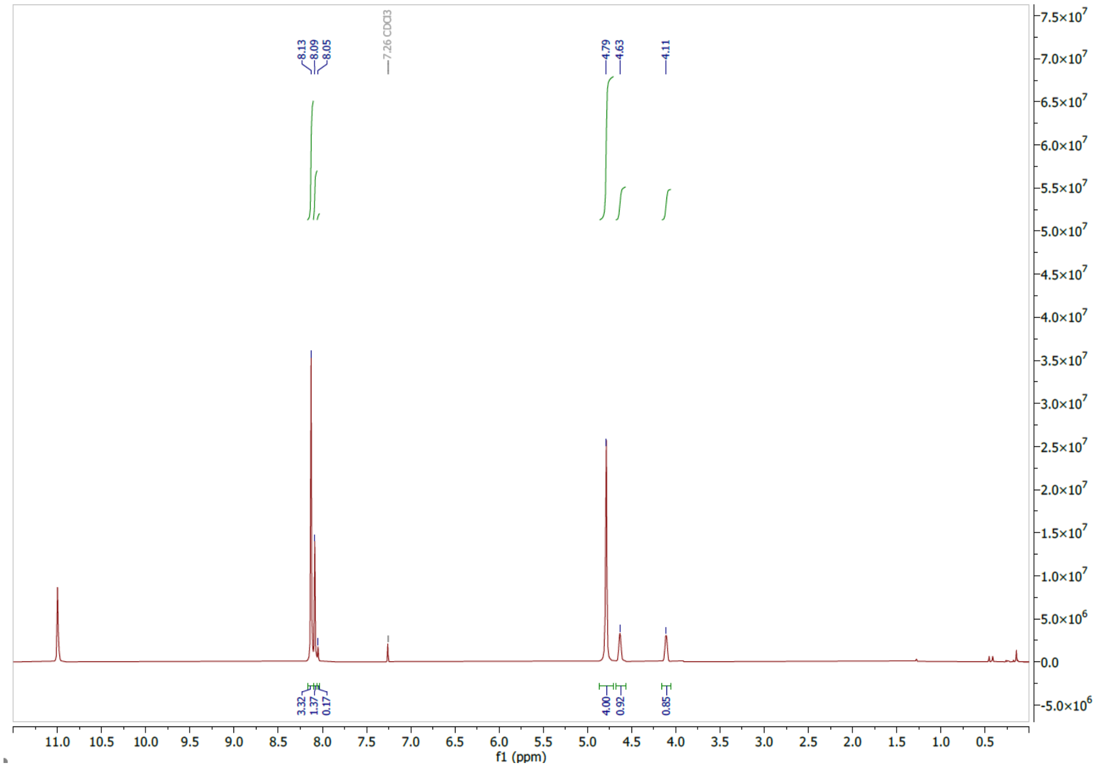

Figure S26: ^{1}H-NMR spectra of rPET produced from crude TPA (top) and rPET produced from TPA washed with ethylene glycol (bottom).

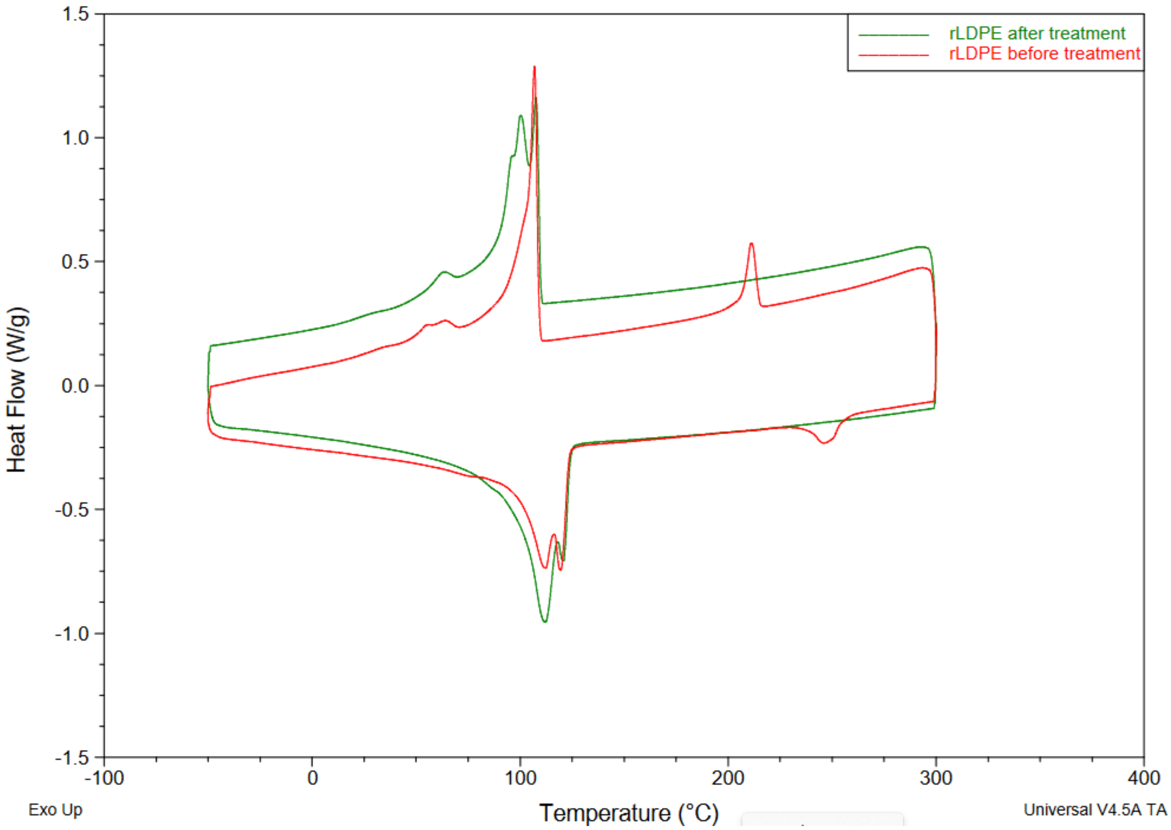

Figure S27. DSC analysis of recovered PE fraction before (red) and after (green) removal of residual PET by alkaline hydrolysis

Table S1: A summary of the setup and results of reported pH-controlled reactions in (bio)reactors. "TPA_eq" is an abbreviation of terephthalic acid equivalents, also encompassing isophthalic acid and MHET. "TPA recovery" is the dry weight of total obtained product as percentage of the expected dry weight based on the yield measured by HPLC. "TPA yield" is the amount of mmol recovered as product as percentage of the amount of mmol TPA introduced as PET substrate.

**Substrate**	**reaction volume (L)**	**salts**	**solids (%)**	**enzyme**	**enzyme loading (mg/mL)**	**time (h)**	**temperature (°C)**	**PET depolymerization (%)**	**TPA_eq HPLC yield (%)**	**TPA recovery (% w/w)**	**TPA yield (%)**	**TPA purity (% w/w)**	**residual PE purity (% w/w)**
GfPET	1	-	1	LCC	0.8	43	70	99	99	-	-	-	-
PET-PE#1	1	-	1	LCC	0.8	67	70	89	91	-	-	-	36.0±3.5
PET-PE#2	1	-	1	LCC	0.8	67	70	84	95	-	-	-	24.0±1.1
PET-PE#1	0.4	-	1.25	LCC	0.8	115	70	80	83	-	-	-	-
PET-PE#1	0.4	-	1.25	LCC	0.8	115	70	94	87	-	-	-	-
PET-PE#2	0.4	-	1.25	LCC	0.8	115	65	97	95	-	-	-	-
PET-PE#2	0.4	-	1.25	LCC	0.8	115	65	96	92	-	-	-	-
PET-PE#2	0.2	1 M NaCl	2.5	PHL7	4.1	139	65	85	90	-	-	-	-
PET-PE#2	0.2	1 M NaCl, 0.2 M NaPi	10	PHL7	0.8	190	65	94	86	94	81	94.9±1.3	52.5±5.4
PET-PE#2	0.2	1 M NaCl, 0.2 M NaPi	20	PHL7	0.8	190	65	96	84	96	80	94.2±0.7	60.7±0.9
PET-PE scrap	90	1 M NaCl, 0.2 M NaPi	5	PHL7	4.0	144	65	98	-	-	-		
PET-PE scrap	90	1 M NaCl, 0.2 M NaPi	5	PHL7	4.0	144	65	99	-	-	-	97	65
PET-PE scrap	90	1 M NaCl, 0.2 M NaPi	5	PHL7	2.0	144	65	95	-	-	-		

Table S2: Molecular weight of PET in residual plastic material obtained after enzymatic hydrolysis of PET in PET-PE#2 using crude PHL7 as determined by GPC.

Sample	M_w (10^3 g/mol)	PET content (% w/w)
PET-PE#2 hydrolysed at 200 g/L plastic	52	35
PET-PE#2 hydrolysed at 100 g/L plastic	52	45
PET-PE#2 starting material	48	97

Table S3: Downstream processing of PHL7 produced in *P. pastoris* in a 300 L bioreactor. The pilot-scale crude PHL7 showed higher specific esterase activity (45 U/mg) than the previous purified PHL7 (23 U/mg). Possible explanations include biological variation (different protein folding, secretion, or glycosylation), assay variability, and enzyme instability during production and/or processing. We have adhered to the specific activity of purified PHL7 for calculating the enzyme loading in milligrams in subsequent reactions, for the sake of comparability.

Downstream steps	Vol.	Activity			Protein
	[L]	[U/mL]	[KU]	[%]	[mg/mL]
Supernatant CEPA	175	48.3	8,449	100	0.89
UF(10KDa) Concentrate	4.0	1,891	7,566	90	42.0
UF (10KDa) Rinse	1.1	152	167	2	2.4
UF (10KDa) Filtrate	170	0.02	3.4	0	17.6

Table S4. Thermal properties of the different PE fractions analysed.

Formulation	ΔHc ($J \cdot g^{-1}$)	T_c (°C)	ΔHm_1 ($J \cdot g^{-1}$)	T_{m1} (°C)	ΔHm_2 ($J \cdot g^{-1}$)	T_{m2} (ºC)
Crude PE from hydrolysate	85	107	82	112; 120	8,5	247
PE from hydrolysate after alkaline treatment	134	108	123	111; 120	-	-
